# Asymmetric diversification of mating pheromones in fission yeast

**DOI:** 10.1371/journal.pbio.3000101

**Published:** 2019-01-22

**Authors:** Taisuke Seike, Chikashi Shimoda, Hironori Niki

**Affiliations:** 1 Genetics Strains Research Center, National Institute of Genetics, Mishima, Shizuoka, Japan; 2 Graduate School of Science, Osaka City University, Sumiyoshi-ku, Osaka, Japan; Duke University Medical Center, UNITED STATES

## Abstract

In fungi, mating between partners depends on the molecular recognition of two peptidyl mating pheromones by their respective receptors. The fission yeast *Schizosaccharomyces pombe* (Sp) has two mating types, Plus (P) and Minus (M). The mating pheromones P-factor and M-factor, secreted by P and M cells, are recognized by the receptors mating type auxiliary minus 2 (Mam2) and mating type auxiliary plus 3 (Map3), respectively. Our recent study demonstrated that a few mutations in both M-factor and Map3 can trigger reproductive isolation in *S*. *pombe*. Here, we explored the mechanism underlying reproductive isolation through genetic changes of pheromones/receptors in nature. We investigated the diversity of genes encoding the pheromones and their receptor in 150 wild *S*. *pombe* strains. Whereas the amino acid sequences of M-factor and Map3 were completely conserved, those of P-factor and Mam2 were very diverse. In addition, the P-factor gene contained varying numbers of tandem repeats of P-factor (4–8 repeats). By exploring the recognition specificity of pheromones between *S*. *pombe* and its close relative *Schizosaccharomyces octosporus* (So), we found that So-M-factor did not have an effect on *S*. *pombe* P cells, but So-P-factor had a partial effect on *S*. *pombe* M cells. Thus, recognition of M-factor seems to be stringent, whereas that of P-factor is relatively relaxed. We speculate that asymmetric diversification of the two pheromones might be facilitated by the distinctly different specificities of the two receptors. Our findings suggest that M-factor communication plays an important role in defining the species, whereas P-factor communication is able to undergo a certain degree of flexible adaptation–perhaps as a first step toward prezygotic isolation in *S*. *pombe*.

## Abstract 2

An asymmetric pheromone/receptor system in the fission yeast Schizosaccharomyces pombe might allow flexible adaptation of pheromones to mutational changes while maintaining stringent recognition for mating partners, perhaps as a first step toward prezygotic mating isolation.

## Author summary

The emergence of a new species might occur when two groups can no longer mate. Although such reproductive isolation is considered a key evolutionary process, the mechanisms by which it actually occurs have been confined to conjecture. The two sexes (Plus [P] and Minus [M]) of *S*. *pombe* each secrete a pheromone (P-factor and M-factor), which binds to a corresponding receptor (mating type auxiliary minus 2 [Mam2] and mating type auxiliary plus 3 [Map3]) on cells of the opposite sex. The interaction between a pheromone and its receptor is essential for successful mating. Here, we explored conservation of the mating pheromone communication system among 150 wild *S*. *pombe* strains of different geographical origins and the closely related species *S*. *octosporus*. We found that 1) the M-factor/Map3 interaction was completely conserved, whereas the P-factor/Mam2 interaction was very diverse in the strains investigated, and 2) most of the P-factor variants were functional across species. Thus, we have revealed an asymmetric pheromone/receptor system in fungal mating: namely, whereas M-factor communication operates extremely stringently, P-factor communication has the flexibility to create variations, perhaps facilitating prezygotic isolation in *S*. *pombe*.

## Introduction

Reproductive isolation, which restricts gene flow between sympatric populations, is one of the key mechanisms of speciation [1]. Mating between individuals of closely related species is prevented by a prezygotic barrier, which is mainly caused by changes in signals that enable individuals to appropriately recognize the opposite sex: for example, pheromones in insects [2,3] and amphibians [4,5], body color in fish [6], and song in birds [7]. Although such reproductive isolation has been frequently studied in higher organisms, far less is known in fungi [8]. In ascomycetes including yeasts, mating between partners critically depends on the molecular recognition of peptidyl mating pheromones by receptors [9,10]. Our recent study in the fission yeast *S*. *pombe* demonstrated that several mutations in a pheromone and its corresponding receptor created a new prezygotic barrier that can give rise to a new species [11]. This experimental observation supports the idea that pheromone/receptor systems drive reproductive isolation through very subtle variations in nature. Thus, genetic alterations of pheromones and their receptors are likely to be important to promote speciation in yeasts. More generally, however, loss of pheromone activity may result in extinction of an organism’s lineage; therefore, changes in the mating pheromone systems might occur gradually and/or coincidently before speciation happens. This hypothesis is an attractive explanation for the speciation process in yeasts, but the mechanisms of genetic alterations of pheromone/receptor systems in nature remain to be elucidated.

*S*. *pombe* has two mating types, Plus (P) and Minus (M) [12,13]. Under nitrogen-limited conditions, two haploid cells of opposite mating types mate via reciprocal stimulation of their mating pheromone receptors [14]. P cells secrete a mating pheromone called P-factor, a simple 23-amino acid peptide, which is recognized by its corresponding G-protein coupled receptor (GPCR) mating type auxiliary minus 2 (Mam2) on M cells [15,16]. The mating type auxiliary plus 2^+^ (*map2*^+^) gene encodes a precursor polypeptide containing four tandem repeats of mature P-factor, which is a mixture of three different peptides in the laboratory strain. P-factor is secreted by the standard secretory pathway [16]. On the other hand, M cells secrete a mating pheromone called M-factor, which is recognized by its GPCR Map3 on P cells [17]. Mature M-factor, a farnesylated and methylated peptide of nine amino acids, is encoded by three redundant genes: *mfm1*^+^, *mfm2*^+^, and *mfm3*^+^ [18,19]. Each of these genes generates a precursor containing a single copy of the same M-factor sequence. M-factor is secreted specifically by the ATP-binding cassette (ABC) transporter Mam1 [20]. *S*. *pombe* M cells also produce a pheromone-degrading enzyme encoded by the sexually activated 2^+^ (*sxa2*^+^) gene [21–23]. Sxa2 is a serine carboxypeptidase that specifically degrades extracellular P-factor. The C-terminal Leu residue of P-factor is removed by Sxa2, which is secreted by M cells [22], and the resulting P-factor lacking Leu is inactive and not recognized by Mam2 [24]. Yeast cells sense a gradient of pheromones secreted by the opposite cell and then extend a mating projection toward the pheromone source [25]. Degradation of the pheromones by the peptidase is thought to make the gradient more stable [26]. In contrast, an enzyme that degrades M-factor has not yet been found. Instead, expression of the *mfm* genes encoding M-factor might be differentially controlled to facilitate fine tuning. These differences in the two mating pheromones, including chemical structure, secretion pathway, and degradation, are widely common in ascomycetes [9], but the biological significance remains unclear.

The standard laboratory strain of *S*. *pombe*, L968, has four copies of the P-factor–encoding sequence and three genes encoding M-factor [27]. Hence, evolution has a mechanism for creating new versions of pheromones, while at the same time, cells retain the ability to mate via the original versions; therefore, we hypothesized that the redundancy in the pheromones might allow unrestricted diversification. In this study, we examined how pheromones and their receptors coevolve in nature. First, we examined pheromone diversity by determining the nucleotide sequences of the pheromone and receptor genes in 150 wild *S*. *pombe* strains [28,29], finding that the amino acid sequence of M-factor and Map3 was completely conserved, whereas that of P-factor and Mam2 was very diverse. In some strains, for example, the copy number of P-factor increased to 5–8 repeats. Second, we analyzed the specificity of pheromones–receptor recognition between *S*. *pombe* and the related species *S*. *octosporus*. Whereas So-M-factor was not functional in *S*. *pombe*, all So-P-factors tested were partially functional in *S*. *pombe*, enabling these cells to mate successfully using So-P-factors. Thus, recognition of M-factor is highly stringent, whereas that of P-factor is relatively relaxed. Our findings suggest that M-factor communication plays an important role in partner discrimination, whereas P-factor communication allows flexible adaptation to create variations. We speculate that such an asymmetric pheromone/receptor system may have evolved to create a prezygotic barrier in *S*. *pombe*.

## Results

### The amino acid sequences of M-factor and Map3 are completely conserved, whereas those of P-factor and Mam2 are diverse

**Fig 1:**
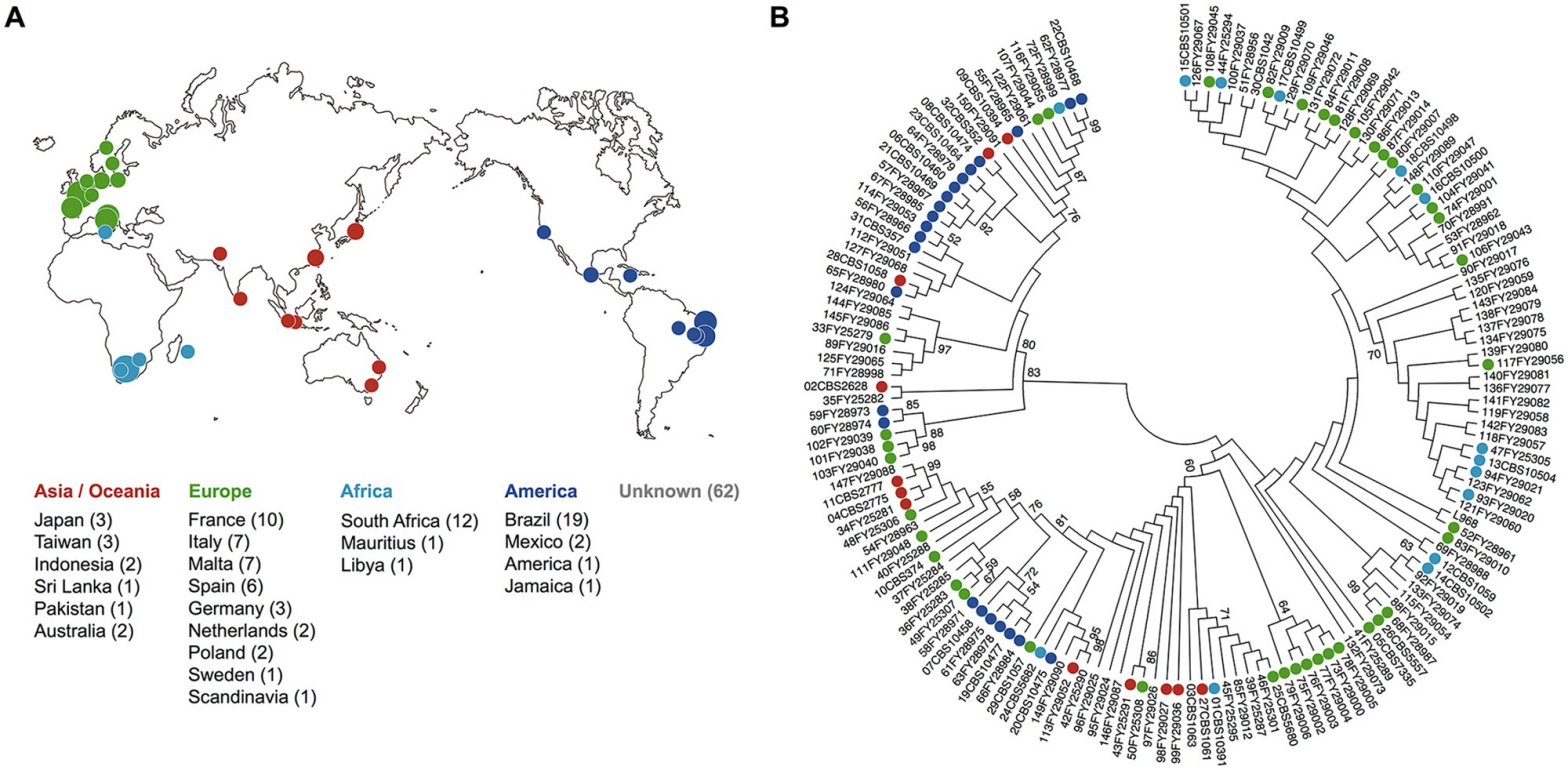
Evolutionary relationship of 150 wild *S*. *pombe* strains based on pheromone/receptor nucleotides. (A) Geographical origins of the 150 wild strains (S2 Table). Colored circles indicate the approximate countries and regions of the isolated strains, which are color-coded by continents: Asia/Oceania, Europe, Africa, and America. Circle size increases in proportion to the number of strains isolated from each region; the actual number of strains is shown in parentheses. Location was unknown for 62 strains. (B) Phylogenetic tree of 151 *S*. *pombe* strains, including the laboratory strain (L968). The analysis involved combined multiple sequence alignments for *mfm1*, *mfm2*, *mfm3*, *map2*, *map3*, and *mam2*. Evolutionary history was inferred by using the Neighbor-Joining [30]. The optimal tree with the sum of branch length = 0.03655487 is shown. The evolutionary distances were computed by using the Kimura 2-parameter method [31] and are shown in units of the number of base substitutions per site. All ambiguous positions were removed for each sequence pair. There were a total of 3,737 positions in the final data set. Evolutionary analyses were conducted in MEGA7 [32]. mam, mating type auxiliary minus; map, mating type auxiliary plus; MEGA, Molecular Evolutionary Genetics Analysis software; mfm, mating factor minus.

To investigate the diversity of *S*. *pombe* mating pheromones and their corresponding receptors in nature, we analyzed 150 wild strains (S1 and S2 Tables) whose origins differ from the standard laboratory strain, L968, first described by U. Leupold [27]. These strains were derived from various countries and regions (Fig 1A) and were isolated from several different sources (S2 Table) [28]. We sequenced the M-factor genes (*mfm1*, *mfm2*, and *mfm3*), the receptor gene for M-factor (*map3*), the P-factor gene (*map2*), and the receptor gene for P-factor (*mam2*) of all 150 strains and compared the nucleotide sequences with that of the L968 strain registered in the database (PomBase, https://www.pombase.org). We constructed a phylogenetic tree based on the sequences of these six genes in the 151 *S*. *pombe* strains (Fig 1B) (trees for the individual genes are shown in S1 Fig). Many nucleotide differences in these genes were found among the strains (S3 and S4 Tables). As shown in Fig 1B, the sequence patterns of these strains were relatively diversified, with characteristic patterns depending, in part, on region (i.e., Europe or South America).

**Fig 2:**
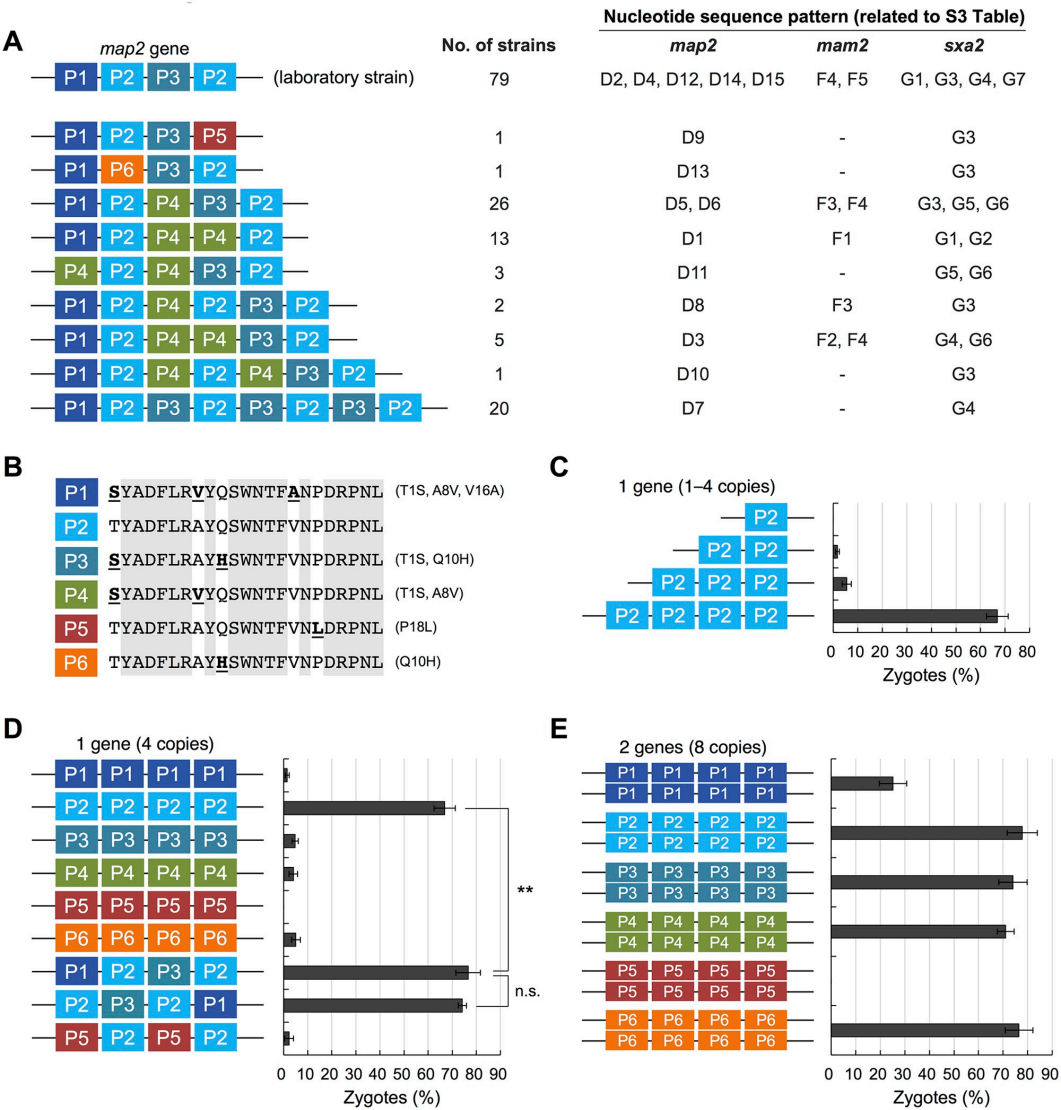
Six different P-factors are found in nature. (A) Diversified pattern of the *map2* gene in nature. In the laboratory strain (L968), the *map2*^+^ gene carries four tandem P-factor–encoding repeats (P1-P2-P3-P2). About half of the 150 wild strains had a *map2* gene with a higher number of P-factor repeats (5–8 repeats). The nucleotide sequence patterns of the *map2*, *mam2*, and *sxa2* genes (see S3 Table) corresponding to each P-factor variant are shown next to the diagram. (B) The six different P-factors (P1–P6) found in nature are shown below. P2 peptide is the standard P-factor of *S*. *pombe*; the amino acids that differ from the P2 peptide are underlined in bold. Identical amino acids are shown in gray. (C) Zygote frequency of strains with a *map2* ORF containing different numbers of P-factor–encoding repeats (i.e., 1–4 identical repeats of the P2 peptide). (D) Zygote frequency of strains producing various P-factors (all with 4 repeats). (E) Zygote frequency of strains expressing two *map2* genes, each with 4 repeats (8 copies in total). At least 300 cells were examined for each sample. Data are the mean ± SD of triplicate samples. The numerical data are included in S2 Data. Statistical significance was assessed by *t* test (**p < 0.01). mam, mating type auxiliary minus; map, mating type auxiliary plus; mfm, mating factor minus; n.s., not significant; ORF, open reading frame; P, Plus; sxa, sexually activated.

Notably, three *mfm* genes of all 151 strains (i.e., 453 genes in total) produced an identical mature M-factor peptide, YTPKVPYMC^Far^-OCH_3_ (102 genes have been previously reported [33]). Many mutations were found in the prosequences and introns, but only one mutation was found in the mature M-factor–encoding region; this was a synonymous change that did not cause an amino acid substitution (S4 Table). Moreover, the amino acid sequences of Map3 in the 150 strains were also identical to that in the L968 strain (Table 1). In other words, the amino acid sequences of the M-factor/Map3 pair seem to be completely conserved in nature. In contrast, the sequences of the *map2* genes were very diverse (S3 and S4 Tables). Whereas the *map2* gene in L968 carries four P-factor–encoding tandem repeats (P1-P2-P3-P2), extensive variations in the number of repeats were observed in the 150 strains, ranging from four to eight (Fig 2A). In addition, the *map2* genes of the wild strains were predicted to produce six different mature P-factor peptides (P1–P6; see Fig 2B). Interestingly, there were five different amino acid sequences of Mam2 across the 150 strains (Table 1). Thus, the amino acid sequences of the P-factor/Mam2 pair seem to be very diverse in nature. Collectively, these findings show that the two mating pheromones in *S*. *pombe* have diversified asymmetrically.

**Table 1:** Summary of polymorphisms of the seven pheromone-associated genes in 151 *S*. *pombe* strains.

Gene	Polymorphism		Nucleotide sequence pattern
*mfm1* (M-factor)	1	WT	WT, A1, A2, A3, A4, A5, A6, A7
*mfm2* (M-factor)	1	WT	WT, B1, B2, B3, B4, B5
*mfm3* (M-factor)	1	WT	WT, C1, C2, C3
*map2* (P-factor)	6	WT (P2)	WT, D1, D2, D3, D4, D5, D6, D7, D8, D9, D10, D11, D12, D13, D14, D15
		T1S, A8V, V16A (P1)	WT, D1, D2, D3, D4, D5, D6, D7, D8, D9, D10, D12, D13, D14, D15
		T1S, Q10H (P3)	WT, D2, D3, D4, D5, D6, D7, D8, D9, D10, D11, D12, D13, D14, D15
		T1S, A8V (P4)	D1, D3, D5, D6, D8, D10, D11
		P18L (P5)	D9
		Q10H (P6)	D13
*map3* (M-factor receptor)	1	WT	WT, E1, E2, E3, E4
*mam2* (P-factor receptor)	5	WT	WT, F4
		I101V	F2
		H155R	F1
		K158N	F5
		T164S	F3
*sxa2* (P-factor–degrading enzyme)	4	WT	WT
		K62E, N82D, Q184E, N208Y, V248M	G1, G2, G3, G5
		K62E, N82D, Q184E, N208Y, V248M, G315D	G4, G6
		K62E, N82D, Q184E, N208Y, V248M, V321I	G7

**Abbreviations**: M, Minus; mam, mating type auxiliary minus; map, mating type auxiliary plus; mfm, mating factor minus; P, Plus; sxa, sexually activated; WT, wild type.

These nucleotide sequence patterns are described in S3 Table.

### Wild *S*. *pombe* strains simultaneously produce multiple P-factor peptides

Having observed increased numbers of P-factor–encoding repeats in the *map2* gene in about half of the 150 strains (Fig 2A), we tested whether repeat number directly affects mating frequency. The native 4-repeat Map2 open reading frame (ORF) from the L968 strain was replaced with an ORF carrying different numbers of the P2 repeat, which was first characterized as P-factor of *S*. *pombe* [16] (see Materials and methods). We found that a decrease in P2 repeat number (<4 repeats) resulted in an extremely low frequency of zygotes (Fig 2C). For example, the strain with the 3-repeat ORF produced less than one-tenth of the zygotes (%) of the strain with the 4-repeat ORF (Fig 2C). However, few significant differences in mating efficiency were observed among the strains carrying an ORF with more than 4 repeats (S2A Fig).

To compare the activity of the different P-factor peptides (P1–P6), we introduced a modified *map2* gene carrying four tandem repeats of each P-factor into the P-factorless strain (FY23418; S1 Table), in which the native *map2*^+^ gene had been deleted (see Materials and methods). The resulting strains each produced one of the six P-factors. The mating efficiency of these strains was assessed by the frequency of zygotes. The strain producing four P2 peptides from the *map2* gene showed a high frequency of zygotes (66.9% ± 4.4%); remarkably, however, the zygote frequency of the remaining strains producing the other P-factor peptides (P1, P3–P6) was extremely low or zero (Fig 2D). Furthermore, in a strain in which the native *map2*^+^ gene carrying a sequence of repeats (P1-P2-P3-P2) was introduced, the frequency of zygotes was fairly high (76.6% ± 5.1%), despite the production of only two copies of the P2 peptide (Fig 2D). To determine whether the order of nucleotide sequences encoding mature P-factor affects zygote frequency, we also introduced a *map2* gene carrying a permuted sequence of repeats (P2-P3-P2-P1) into the FY23418 strain. Similar to the strain with the native sequence, this strain also mated at high frequency (74.2% ± 1.7%) (Fig 2D); therefore, the effect of peptide-coding position appears small.

Next, we considered that if mature P-factor production is modulated by a length-dependent biosynthetic pathway, the low mating efficiency observed in strains carrying an ORF with fewer than 4 repeats might be attributed to translation level. To examine this possibility, we constructed a *map2* gene carrying the four tandem P-factor–encoding repeats P5-P2-P5-P2 and introduced it into the FY23418 strain. Surprisingly, however, this strain was almost as sterile as the strain with the 2-repeat ORF (Fig 2C and 2D). Thus, the apparently inactive P1 and P3 peptides might have important roles during the mating process. The amino acid sequences of the P1 and P3 peptides slightly differ from that of the P2 peptide (Fig 2B). In fact, some peptides with slight mutations of P1–P3 had markedly decreased mating efficiency (S2B Fig). These results indicate that the substitution of only a few amino acids of P-factor have a large influence on copulation.

To investigate further the causes of the low frequencies of the P-factor peptides (P1, P3–P6), we constructed strains carrying two *map2* genes encoding four tandem repeats of each P-factor (i.e., eight copies in total) and examined the zygote frequency of these strains in the same way. Remarkably, the strains producing eight copies of peptide showed a much higher frequency of zygotes (Fig 2E) relative to those carrying one gene (Fig 2D). We considered that this is probably due to a dose-dependent effect. Overall, these results indicated that the P2 peptide is likely to be most compatible with the native Mam2 receptor; curiously, however, no wild strains that we investigated generated a precursor containing only copies of the P2 peptide sequence.

### Production of multiple P-factors might be advantageous for mate choice

As described above, the *map2* gene was found to contain at least four tandem repeats encoding multiple P-factor peptides in all 150 wild strains. To assess the effect of producing multiple P-factors, we performed a quantitative competitive mating assay. In this experiment, P cells producing a multiple P-factor (P1-P2-P3-P2) and P cells producing a single P-factor (P2-P2-P2-P2) were inoculated with wild-type M cells onto malt extract agar (MEA) plates at a cell number ratio of 1:1:2. The three strains were differentially marked by different drug-resistant markers (see Materials and methods). After incubation for 24 hours, the cell suspension was spread onto yeast extract agar (YEA) plates containing combinations of the appropriate drugs. Doubly resistant hybrid descendants of mating between P cells and M cells were counted to determine the recombinant frequency.

**Fig 3:**
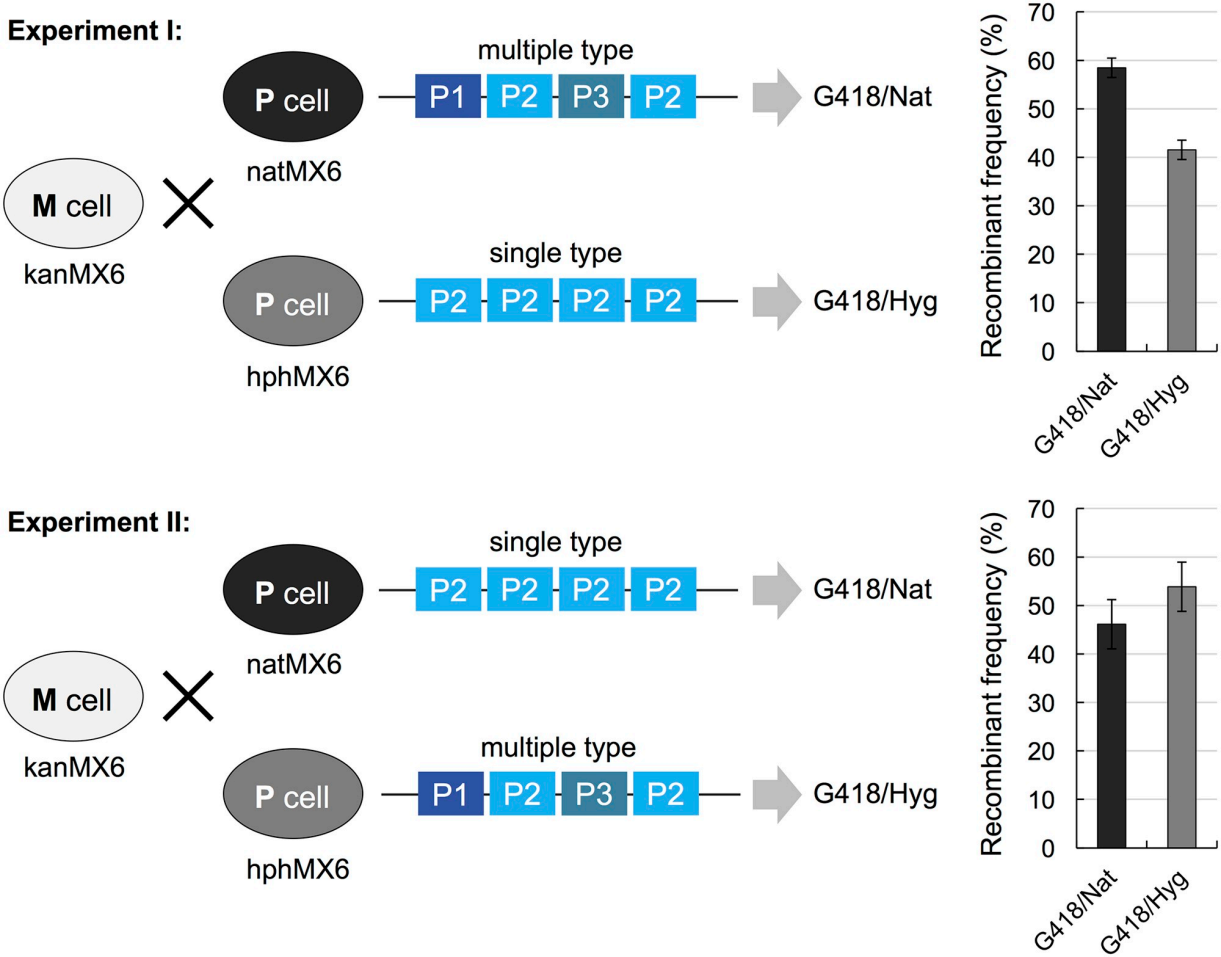
Recombinant frequency of strains producing a multiple or single type of P-factor. The assay was conducted by using heterothallic haploid strains carrying a kanMX6, hphMX6, or natMX6 drug-resistant marker as follows: Experiment I, wild-type M cells (FY23362; kanMX6), P cells producing a multiple-type P-factor (TS368; natMX6), and P cells producing a single-type P-factor (TS232; hphMX6); Experiment II, wild-type M cells (FY23362; kanMX6), P cells producing a single-type P-factor (TS59; natMX6), and P cells producing a multiple-type P-factor (TS369; hphMX6). The same number of two P cells and twice the number of cells of the M cells were mixed and grown on MEA. After incubation for 1 day, the cultures were diluted and then spread onto YEA plates containing 100 μg/ml of the following drugs: YEA+G418/Nat and YEA+G418/Hyg. After 3 days of incubation, the YEA plates were photographed. The colony numbers on plates were counted, and the recombinant frequency was calculated. Data are the mean ± SD of three independent experiments. The numerical data are included in S2 Data. M, Minus; MEA, malt extract agar; P, Plus; YEA, yeast extract agar.

The assay indicated that M cells showed a slight preference to mate with P cells producing a multiple-type P-factor as compared with a single-type P-factor (Fig 3). This tendency was not influenced by the type of drug-resistant marker used (Fig 3). These results suggest that the production of multiple P-factors might be advantageous for mate choice by M cells.

### Most P-factor peptides are recognized by Mam2

In *S*. *pombe*, P-factor is largely degraded outside the cell by the carboxyl peptidase Sxa2 [22]. Next, therefore, we examined whether each P-factor peptide is truly recognized by Mam2 in the absence of Sxa2. An M-type strain lacking Sxa2 (TS402; S1 Table) was treated with synthetic P-factors at different concentrations (0–1,000 nM) in nitrogen-free liquid medium (EMM2−N). Yeast cells elongate a mating projection (“shmoo”) when they sense sufficient pheromones; therefore, the ability of Mam2 to recognize the different P-factors was determined by measuring the ratio between the length (L) and width (W) of an individual cell (see Materials and methods). Here, we defined cells with an L/W ratio of 2.0 or more as shmooing cells.

The addition of P2 peptide clearly induced cell elongation at 10 nM after 1 day of incubation. According to our definition, one-third of cells treated with P2 peptide (10 nM) became shmooing cells in response to P-factor after 1 day (S3 Fig). Notably, cells treated with 10 nM P1, P3, P4, and P6 also elongated, indicating that these peptides were recognized by Mam2. As compared with P2 peptide, however, the proportion of shmooing cells after treatment with these peptides was significantly low (15%–23% at 10 nM; see S3 Fig). In contrast, the P5 peptide, produced only by the 24CBS5682 strain (S2, S3 and S4 Tables), was not recognized by Mam2 even when the cells were treated with a concentration of 1,000 nM (S3 Fig). To measure the activity of the P5 peptide accurately, we used a P-factor–sensitive strain (TS578; see Materials and methods) and treated the cells with 1,000 nM synthetic P5 peptide. As shown in S4 Fig, no cells underwent shmoo elongation. This result indicates that the P5 peptide is not recognized by native Mam2, as far as we have examined. In conclusion, the shmoo formation assay indicated that most P-factor peptides are sufficiently recognized by Mam2 in vitro.

### P2 peptide is degraded more rapidly by Sxa2

Sxa2 removes the C-terminal Leu residue of P-factor [22]. Based on the above results, we considered that differences in the processing of each P-factor peptide by Sxa2 might affect pheromone-based mate choice. To examine this possibility, the *sxa2*^+^ ORF was cloned into the pTS111 plasmid downstream of the no message in thiamine 1^+^ (*nmt1*^+^) promoter, which is strongly expressed in absence of thiamine [34] (see Materials and methods). The resulting plasmid (pTS284) was then introduced into the TS402 strain lacking Sxa2. The resulting cells were grown in EMM2 medium without thiamine to induce the *nmt1*^+^ promoter, and the culture supernatant was assayed to confirm carboxypeptidase activity (see Materials and methods). Next, each P-factor peptide (200 μM) was mixed with an aliquot of the cell-free culture supernatant including abundant active Sxa2 (total protein 100 ng) for up to 60 minutes, and the amount of leucine released from each P-factor was determined as a measure of Sxa2 activity.

All six P-factor peptides (P1–P6) were sufficiently degraded after 60 minutes in the presence of Sxa2 (Table 2). Unexpectedly, degradation of P2 peptide was found to be more efficient than that of other P-factor peptides. In 10 minutes, approximately 70% of P2 peptide (133.0 ± 21.7 μM) was degraded, as compared with, for example, approximately 50% of P1 (97.7 ± 5.8 μM; see Table 2). These results suggest that native Sxa2 might not efficiently degrade P-factor peptides containing a few residues that differ from P2 peptide. Thus, the P2 peptide is likely to be the best substrate for Sxa2 protease. In fact, *sxa2* deletion mutants are virtually sterile [23]. Hence, degradation of P-factor seems to be important for the correct orchestration of mating. We speculate that the diversification of P-factor probably affects the selection of mates by M cells.

**Table 2:** Amount of leucine released from P-factor by Sxa2 of *S*. *pombe*.

Species	Peptide	Leucine (μM)	
		10 min	60 min
*S*. *pombe*	P1	97.7 ± 5.8	187.0 ± 8.7
	P2	133.0 ± 21.7	200.7 ± 11.6
	P3	117.7 ± 13.2	182.3 ± 10.2
	P4	117.3 ± 18.6	180.7 ± 21.5
	P5	82.0 ± 15.0	196.3 ± 23.1
	P6	115.7 ± 17.5	193.0 ± 25.1
*S*. *octosporus*	P1’	9.7 ± 10.7	63.7 ± 8.1
	P2’	17.7 ± 9.1	83.7 ± 4.0
	P3’	15.0 ± 7.0	69.7 ± 8.3
	P4’	2.7 ± 10.3	49.0 ± 7.0
Control	P2-Leu	1.7 ± 0.4	6.2 ± 0.8

The extent of degradation of each P-factor was determined by the amount of leucine released. Synthetic P-factor peptide (200 μM) was mixed with cell-free culture supernatant (total protein 100 ng), either containing active Sxa2 (TS407 [Sxa2^+^]) or lacking Sxa2 (TS406 [Sxa2^−^]) in 50 mM citrate buffer, pH 5.5. All reactions were performed at 30 °C for the indicated times (10 and 60 minutes) with gentle shaking and were stopped by 0.5% trifluoroacetic acid. Leucine was measured by a branched-chain amino acids (leucine) kit. Data that were calculated by taking a difference between two values in the presence/absence of Sxa2 are the mean ± SD of at least triplicate samples. The numerical data are included in S2 Data. “P2-Leu” is a 22-amino acid peptide whose C-terminal Leu residue of P2 was removed.

**Abbreviation**: sxa, sexually activated.

### Asymmetric diversity of two pheromones is seen in closely related species

As described above, the amino acid sequences of M-factor were completely conserved, whereas those of P-factor were diversified in 150 wild *S*. *pombe* strains analyzed. To determine whether this asymmetry in pheromone diversity is common to other species, we analyzed the nucleotide sequences of both M-factor and P-factor genes in *S*. *octosporus*, the species most closely related to *S*. *pombe*.

**Fig 4:**
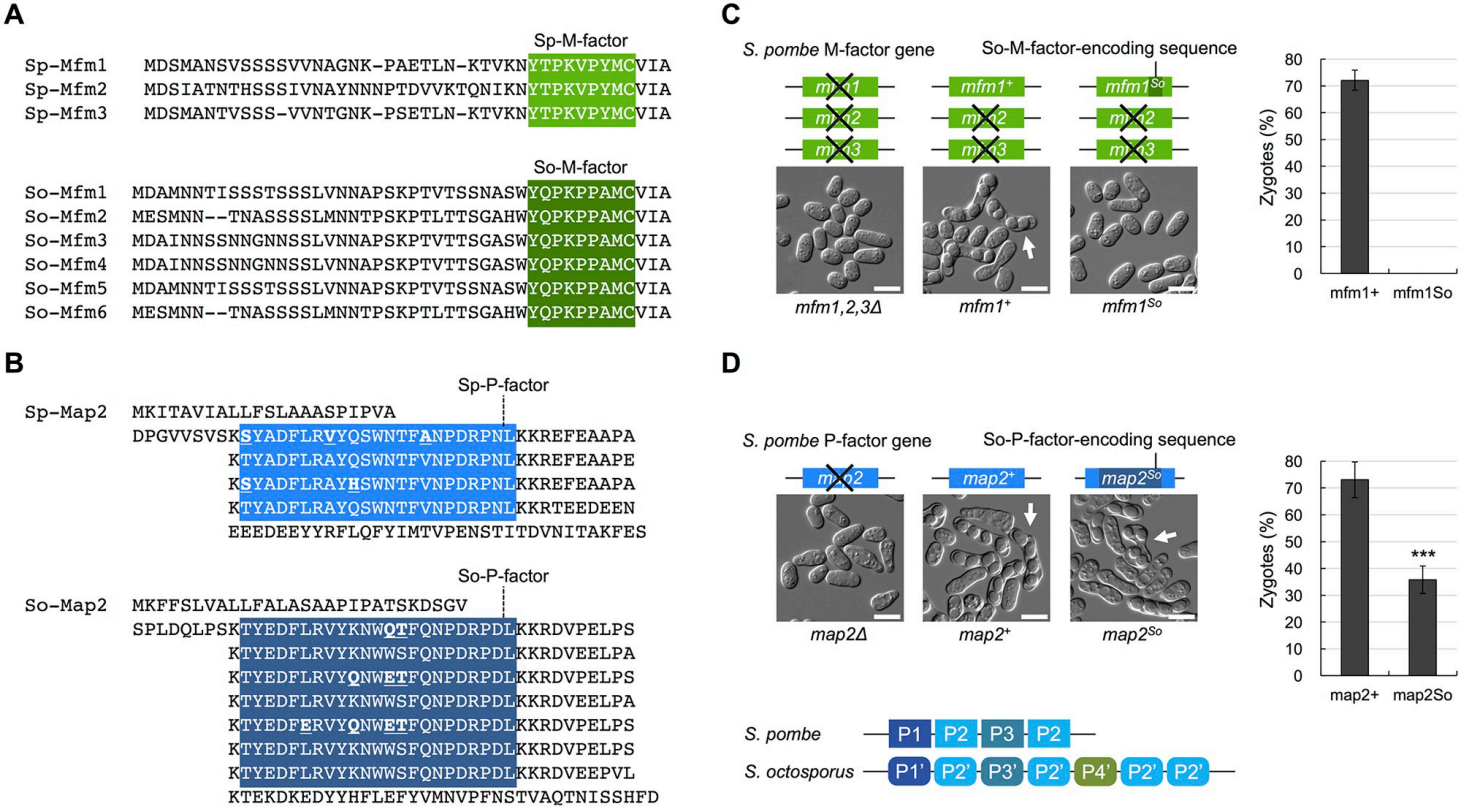
Comparison of mating pheromones between *S*. *pombe* and *S*. *octosporus*. (A) Amino acid sequence alignment of precursors derived from the redundant genes encoding M-factor in *S*. *pombe* (3 genes) (top) and So-M-factor in *S*. *octosporus* (6 genes) (bottom). M-factor precursor proteins of *S*. *octosporus* are tentatively termed So-Mfm1–So-Mfm6. (B) Amino acid sequence alignment of precursors derived from the gene encoding P-factor in *S*. *pombe* (top) and So-P-factor in *S*. *octosporus* (bottom). The P-factor precursor protein of *S*. *octosporus* is tentatively termed So-Map2. The native *So-map2* gene contains seven tandem So-P-factor–encoding repeats (P1’-P2’-P3’-P2’-P4’-P2’-P2’). Multiple sequence alignment was done by a standard algorithm. The mature peptide sequences are indicated by white letters on a color background. The P2’ peptide is the standard So-P-factor of *S*. *octosporus*; amino acids that differ from the standard peptides are underlined in bold. (C) Mating efficiency of the *S*. *pombe* strain producing only So-M-factors. The strain in which the native *mfm1*^+^ gene was replaced with the *mfm1*^*So*^ gene was sterile. (D) Mating efficiency of the *S*. *pombe* strain producing only So-P-factors. The strain in which the native *map2*^+^ gene was replaced with the *map2*^*So*^ gene showed partial fertility (zygotes [%), 35.8% ± 5.1%), as compared with the wild type (zygotes [%], 73.1% ± 6.7%). Data are the mean ± SD of triplicate samples. The numerical data are included in S2 Data. Statistical significance was assessed by *t* test (***p < 0.001). Typical images of mating cells (arrows, tetrad) are shown. Scale bar 5 μm. M, Minus; map, mating type auxiliary plus; mfm, mating factor minus; P, Plus; So, *Schizosaccharomyces octosporus*; Sp, *Schizosaccharomyces pombe*.

Whole-genome sequences of *S*. *octosporus* have been determined by the Broad Institute [35] and indicate that this species has six M-factor–encoding genes (hereafter called “*So-mfm1*–*So-mfm6*”) and one P-factor–encoding gene (hereafter called “*So-map2*”). The six redundant genes (*So-mfm1*–*So-mfm6*) encode M-factor peptides with the same amino acid sequence (Fig 4A); therefore, the primary structures of the putative So-M-factors are the same, YQPKPPAMC^Far^-OCH_3_ (S5A Fig). In contrast, the *So-map2* gene carries seven tandem So-P-factor repeats, which encode four different So-P-factors (Fig 4B and S5C Fig), similar to *S*. *pombe*. Interestingly, therefore, the two pheromones have also diversified asymmetrically in this closely related species.

### P-factor is interchangeable between fission yeast species, but M-factor is not

**Fig 5:**
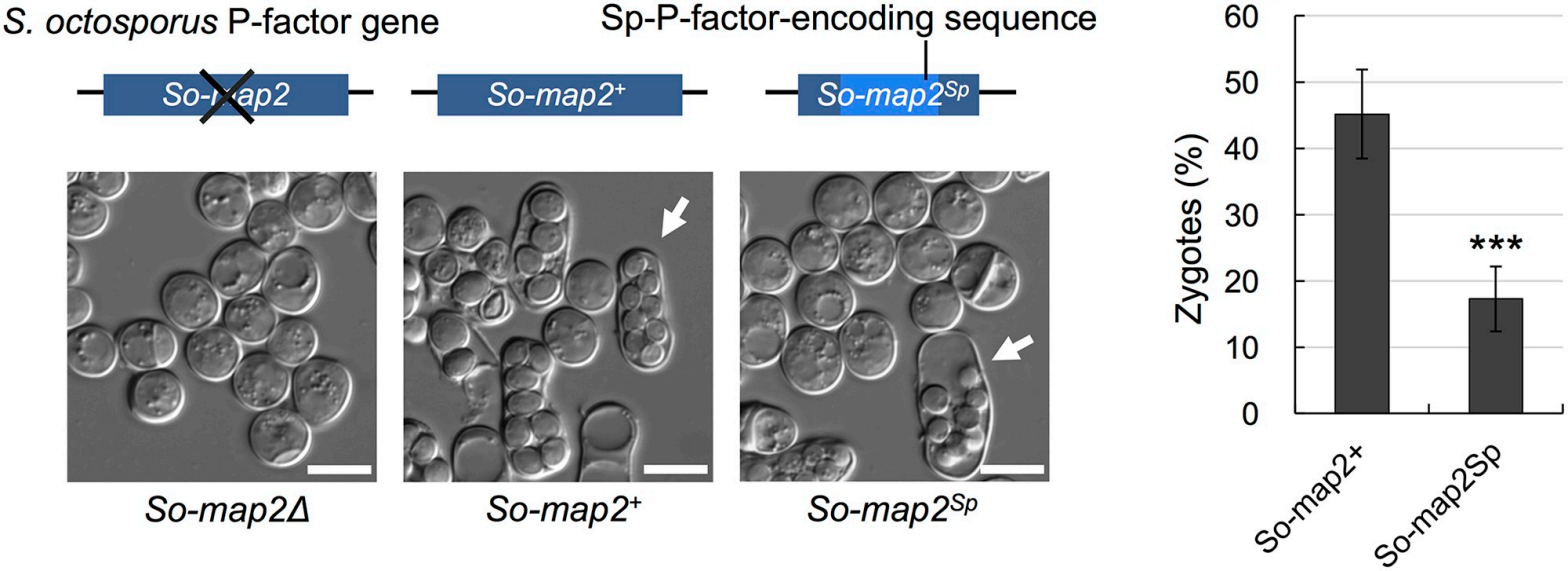
Effects of P-factors on *S*. *octosporus* cells. Mating efficiency of an *S*. *octosporus* strain producing only P-factors was determined by the frequency of zygotes. The strain in which the native *So-map2*^+^ gene was replaced with the *So-map2*^*Sp*^ gene showed partially fertility (zygotes [%), 17.3% ± 4.9%), but fertility was less than the strain with the wild-type gene. Data are the mean ± SD of triplicate samples. The numerical data are included in S2 Data. Statistical significance was assessed by *t* test (***p < 0.001). Typical images of mating cells (arrows, octad) are shown. Scale bar 10 μm. map, mating type auxiliary plus; P, Plus; So, *Schizosaccharomyces octosporus*; Sp, *Schizosaccharomyces pombe*.

Next, we assessed whether the pheromone peptides of *S*. *octosporus* are effective on *S*. *pombe* cells. First, the wild-type *S*. *pombe mfm1*^+^ gene was integrated into the genome of an M-factorless strain (FY23412; S1 Table), which led to high mating efficiency (72.1% ± 3.7%) (Fig 4C). Next, we replaced the Sp-M-factor–encoding sequence in the *mfm1*^+^ gene with the So-M-factor–encoding sequence (*mfm1*^*So*^). The resulting strain, which produced only So-M-factor instead of Sp-M-factor, was found to be completely sterile (Fig 4C). In short, *S*. *pombe* P cells were unable to mate with M cells producing So-M-factor. In contrast, replacement of the Sp-P-factor–encoding sequence (4 repeats) in the *map2*^+^ gene with the So-P-factor–encoding sequence (7 repeats) (*map2*^*So*^), resulted in a strain with approximately half the mating frequency of the strain with the *map2*^+^ gene (35.8% ± 5.1%) (Fig 4D). Thus, *S*. *pombe* M cells can mate with P cells that produce So-P-factors, indicating that at least one of the So-P-factors is effective on *S*. *pombe* cells. We also replaced the So-P-factor–encoding sequence (7 repeats) in the *So-map2*^+^ gene with the Sp-P-factor–encoding sequence (4 repeats) (*So-map2*^*Sp*^) in *S*. *octosporus* cells. The resulting strain, which produced only Sp-P-factor instead of So-P-factor, retained mating ability (17.3% ± 4.9%; see Fig 5). Thus, at least one of the Sp-P-factors is also effective on *S*. *octosporus* cells.

Three of the nine amino acids of So-M-factor were found to differ from those of Sp-M-factor (S5A Fig). To assess whether So-M-factor is recognized at all by the M-factor receptor of *S*. *pombe* (Sp-Map3), we carried out a shmooing assay (see S5B Fig) in which we measured the L/W ratio of cells treated with synthetic So-M-factors at various concentrations in EMM2−N. The M-factor-sensitive strain (TS405; S1 Table) significantly induced cell elongation after one day of incubation with 100 nM Sp-M-factor but showed no elongation when treated with So-M-factor (S5B Fig). Under these conditions, approximately 40% of cells treated with Sp-M-factor peptide underwent shmoo formation, whereas no cells treated with So-M-factor peptide underwent shmooing (S5B Fig). When the concentration of So-M-factor was raised to 1,000 nM, the cells underwent shmoo elongation a little (S5B Fig). These results imply that *S*. *pombe* is reproductively isolated from *S*. *octosporus*, owing to the lack of compatibility of M-factor.

On average, eight of the 23 amino acids of P-factor differed between the two species. To test whether the *S*. *octosporus* P-factor peptides (hereafter named P1’–P4’; S5C Fig) are recognized by the P-factor receptor of *S*. *pombe* (Sp-Mam2), we treated TS402 with each of the four synthetic So-P-factor peptides. Remarkably, cells formed visible shmoos in all cases (S5D Fig). The most effective peptide was P2’; that is, one-fifth of *S*. *pombe* cells treated with P2’ peptide (10 nM) elongated a shmoo in response to P-factor after one day, and the proportion of shmooing cells increased to about 80% at a peptide concentration of 100 nM (S5D Fig). In contrast, recognition of the P4’ peptide by Sp-Mam2 was relatively low. Taken altogether, these findings indicate that all of the So-P-factors have a partial effect on *S*. *pombe* M cells.

### A pheromone-degrading enzyme might facilitate pheromone diversity

The above findings showed that *S*. *pombe* cells can respond to So-P-factors produced by *S*. *octosporus*. We therefore examined whether the P-factor–degrading enzyme of *S*. *pombe* (Sp-Sxa2) can degrade So-P-factors. Each of the synthetic So-P-factor peptides was mixed at 200 μM with culture supernatant containing active Sp-Sxa2, and the amount of leucine released from each P-factor was measured at fixed time points, as described above for Sp-P-factors. All four So-P-factor peptides (P1’–P4’) were considerably degraded in the presence of Sp-Sxa2 (Table 2). The P2’ peptide was degraded most efficiently, with 83.7 ± 4.0 μM degraded after 60 minutes of incubation; as compared with Sp-P-factor peptides, however, Sp-Sxa2 inefficiently removed the Leu residue from So-P-factor peptides (Table 2). In conclusion, Sp-Sxa2 acts most effectively on Sp-P-factors but has the capacity to degrade P-factor peptides from related species such as *S*. *octosporus*, probably limiting the interspecies mating response.

Lastly, we examined the diversity of Sxa2 in nature. In the 150 wild *S*. *pombe* strains, there were four different amino acid sequences of Sxa2 (Table 1 and S4 Table). In exploring the asymmetric diversification of the two mating pheromones, it is notable that M-factor is not degraded by a specific enzyme. We speculate that overlapping regions of P-factor (probably the C terminus) are recognized by both its receptor Mam2 and its degradation enzyme Sxa2, thereby facilitating coevolution of their substrate specificities together with divergence of P-factor. Taken together, diversification of the two pheromones might be accelerated asymmetrically by the distinctly different specificities of the two receptors, in addition to the existence of Sxa2.

## Discussion

Mutational alterations of the pheromone/receptor system can affect the recognition between mating partners, resulting in prezygotic isolation. In this study, we explored the diversification of the pheromones and cognate receptors of *S*. *pombe* in nature. We found that the amino acid sequences of M-factor and its receptor Map3 are completely conserved, whereas those of P-factor and its receptor Mam2 are very diversified (Table 1). Such asymmetric diversification of the two mating pheromones was also seen in the related species *S*. *octosporus* (Fig 4A). Moreover, we noticed that So-M-factor was not functional on *S*. *pombe* cells, whereas all of the So-P-factors tested were partially functional; in other words, *S*. *pombe* cells were capable of mating with cells producing only So-P-factors (Fig 4D) and vice versa (Fig 5). Therefore, it is more likely that the recognition specificities of the two pheromone receptors vary in strictness for their respective pheromones. Map3 and Mam2 are both class IV GPCRs but differ clearly in their amino acid sequences. Recently, Rogers and colleagues [10] reported that a-type pheromones (lipid peptide) either promote efficient mating completely or do not promote it at all, while α-type pheromones (simple peptide) show a more graded distribution of mating efficiency in *Saccharomyces cerevisiae*. Thus, the different specificities of the two GPCRs might lead to asymmetric competence and diversification of pheromones.

*S*. *pombe* has three genes encoding Sp-M-factor, and *S*. *octosporus* has six putative genes encoding So-M-factor (Fig 4A). Such redundancy might enable the cells to alter one copy of M-factor to adapt to genetic changes in Map3, while keeping the others unchanged. In fact, the N-terminal half of M-factor has been shown to be dispensable for recognition by Map3 [33]. Nevertheless, the redundant genes encoding M-factor peptides generate the same sequence of amino acids in nature (Fig 4A). Wild-type *S*. *pombe* has four copies of P-factor, so in principle both pheromones should be free to evolve. We speculate that the amount and/or activity of M-factor might affect mating frequency. Previously, Nielsen and colleagues [19] reported that any one of the three genes of M-factor is sufficient for mating. However, this might not reflect the actual situation in nature. For example, efficient copulation might depend largely on the number of M-factor genes under more severe nutrient-limited conditions, resulting in restricted variation. Because M-factor is encoded by different genes, rather than by repeats, intergenic conversion can occur with considerable frequency [36]. Concerted evolution of the *mfm* genes might allow conservation of the sequence of mature M-factor and the rapid spread of favorable mutations. We could not confirm whether there is an increase or decrease in *mfm* genes in the 150 wild *S*. *pombe* strains, but the reason why the M-factor sequence is fixed will be investigated in a future study.

In the wild *S*. *pombe* strains, the number of P-factor–encoding repeats in the *map2* gene varied from four to eight (Fig 2A). Variations in repeat number in the *Saccharomyces* genus have previously been reported [37,38]. For example, decreasing numbers of repeats in Mfα1, a structural gene for α-factor pheromone, results in a stepwise decrease in α-factor production in *S*. *cerevisiae* [39]. Our experimental data also revealed that removing a single repeat of the 4-repeat sequence in *map2* has a marked effect on mating frequency (Fig 2C). Perhaps, decreasing numbers of repeats in the *map2* gene might lead to a lower production of P-factor in *S*. *pombe*. In addition, a previous study suggested that the secretion pathway has a minimum size requirement for transportation [40]. Hence, the product of the *map2* gene might need to have sufficient length for processing by the biosynthetic pathway. Although a higher repeat number in the *map2* gene might result in increased P-factor production, further increases in repeat number do not necessarily lead to greater pheromone production because Rogers and colleagues [38] also showed that an 8-repeat strain is less favorable than a 6-repeat strain for mating choice in *S*. *cerevisiae*, probably due to reduced pheromone production caused by a decrease in the rate of translation. When the various *map2* genes (4–8 repeats) obtained from the 150 wild strains were integrated into the FY23418 strain, almost all of the resulting strains showed high mating frequency (S2A Fig). The only exception was the *map2* gene (6 repeats) obtained from the 22CBS10468 strain (*map2_D8*; S3 Table), in which a spacer sequence located between the fourth and the fifth repeat was changed from KKR to KKC (S4 Table). The kexin-related endopeptidase kexin-related protease 1 (Krp1) cleaves the KKR motif (three basic amino acids) in the Golgi during the biosynthetic pathway to generate mature P-factor [41]; therefore, such a mutation might affect the production of P-factor. Yeast cells choose a favorable partner producing the highest levels of pheromone, whereas a cell that cannot produce pheromones is not chosen as a mating partner by the opposite mating cell [42,43]. Such fluctuations in the repeat number of P-factor are likely to have an influence on various factors related to mating events.

All of the wild *S*. *pombe* strains produced at least three different P-factors, as far as we investigated (Fig 2A). Curiously, however, our experimental data clearly showed that the P2 peptide was much more efficiently recognized by Mam2 as compared with the others in vivo and in vitro (Fig 2D and S3 Fig). Why don’t *S*. *pombe* cells produce only P2 peptides? Interestingly, we found that cells producing multiple different P-factors seemed to be slightly preferred as a mate by the opposite cell type (Fig 3). This might be explained by the differences in the extent of degradation by Sxa2. In fact, *sxa2* deletion mutants are virtually sterile [23]. Recent studies by Martin’s group have clearly shown that the local pheromone gradient rather than the absolute pheromone concentration is important for efficient mating [26,44]. In this study, we revealed that all constructed strains lacking the *sxa2*^+^ gene showed fairly low zygote frequency (S2D and S6 Figs), and although the P2 peptide is recognized more efficiently by native Mam2, it is also degraded more rapidly by Sxa2 (Table 2). Therefore, we consider that the production of different peptides might affect the whole orchestration of mating by slightly changing the substrate specificity of Sxa2, resulting in the diversification of P-factor.

One of the possible reasons for the extremely low mating frequency of cells expressing each of the P1, P3, P4, and P6 (4 repeats) peptides (Fig 2D) is that these peptides are not expressed as effectively as P2. However, we could not examine this possibility here. All functions of sexual differentiation (i.e., expression of pheromone and receptor genes) are stimulated only by nitrogen starvation in *S*. *pombe*; hence, it is extremely difficult to transfer the majority of conventional methods used with *Saccharomyces* (e.g., Halo assay) to *S*. *pombe*. Nielsen’s group developed an alternative technique based on the observation that pheromone stimulation is necessary to undergo meiosis and sporulation [45]. In our case, however, there are significant differences in bioactivity among the peptides; thus, it is hard to estimate the amount of each P-factor secreted. Therefore, a method that quantifies pheromone secretions independent of the bioactivity of peptides will be needed in *S*. *pombe*. Nevertheless, we believe that the P1, P3, P4, and P6 peptides are certainly expressed because the strains carrying two *map2* genes showed a much higher frequency of zygotes (Fig 2E) relative to those carrying one gene (Fig 2D).

We further noticed that there is a positive relationship between the recognition of P-factor by Mam2 and its efficient degradation by Sxa2 (S3 Fig and Table 2). This is because both Mam2 and Sxa2 are likely to depend on the same regions of P-factor activity. A recent study also suggests that coevolution of sterile 2 (Ste2) (a receptor for α-pheromone) and barrier 1 (Bar1) (a peptidase of α-pheromone) can occur, together with evolution of α-pheromone in *Candida albicans*, because Ste2 and Bar1 recognize the overlapping regions of α-pheromone [46]. In this study, although we did not obtain conclusive evidence that novel compatible combinations of the P-factor/Mam2 and P-factor/Sxa2 pairs can occur (S2C and S2D Fig), such coevolution might proceed little by little, even though reproductive isolation would not be prevented during this time.

**Fig 6:**
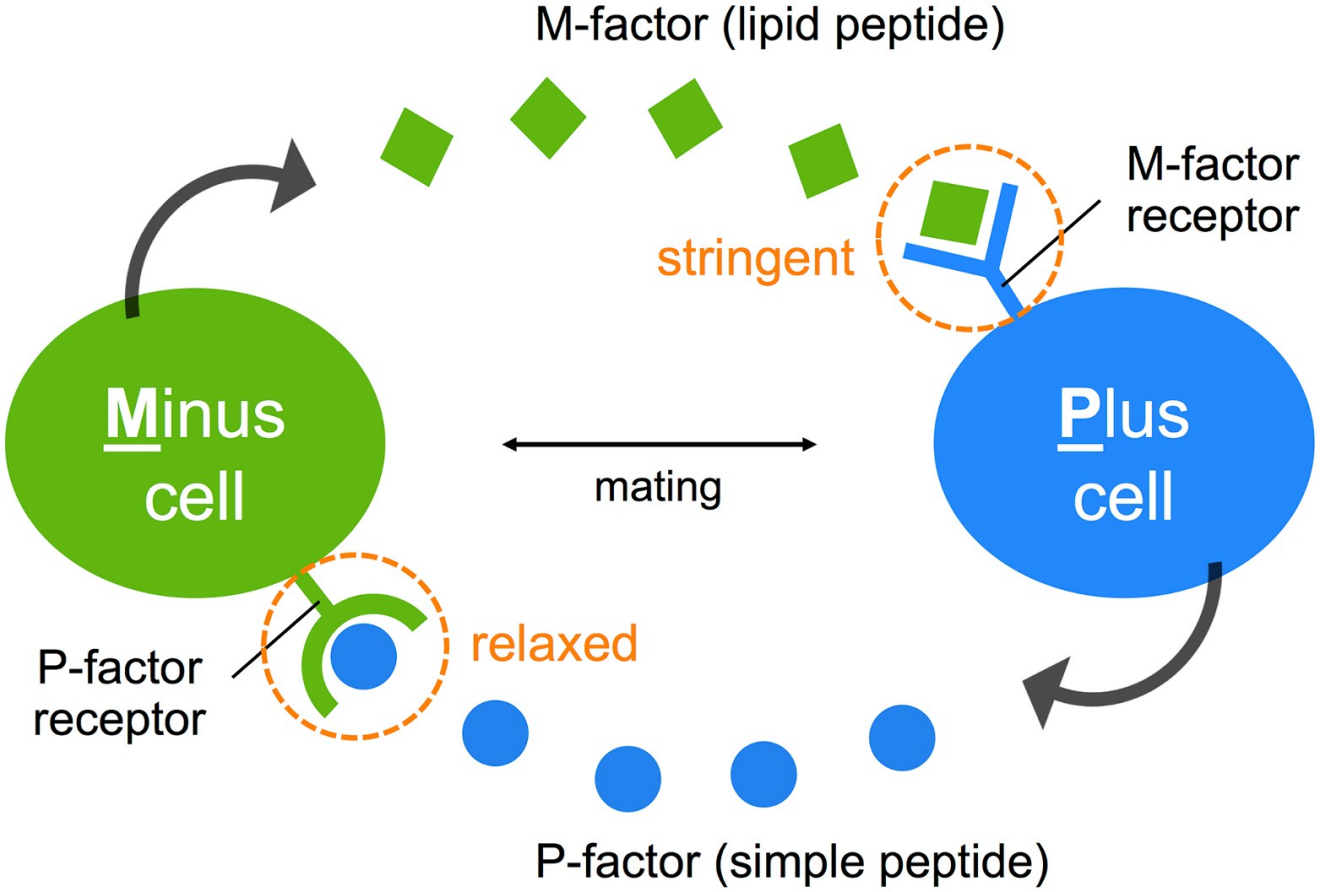
An asymmetric system of pheromone recognition in fission yeast. The specificity of recognition of M-factor (lipid peptide) is extremely stringent, whereas that of the P-factor (simple peptide) is relatively relaxed, allowing cross-reactions to occur between two yeast species. Therefore, the diversity of the two mating pheromones might facilitate asymmetrically in nature. This possession of distinctly different specificities for the mating pheromones might guarantee not only stringent recognition of mating partners but also flexible adaptation to various mutational changes in a combination of a pheromone/receptor. M, Minus; P, Plus.

This study has revealed an asymmetric diversification of the pheromone/receptor system in *S*. *pombe*: namely, recognition by M-factor is extremely stringent, whereas that by P-factor is relatively relaxed (Fig 6). In ascomycetes, one pheromone is a farnesylated peptide with hydrophobicity (i.e., M-factor), while the other is an unmodified peptide with hydrophilicity (i.e., P-factor) [9]. This chemical asymmetry might be more beneficial for yeasts living in a liquid environment because a hydrophilic peptide is probably more diffusible and therefore might reach far-away cells, enabling rapid identification of mating partners. In fact, our findings lead us to propose two hypotheses. One is that the sexual behavior of individual species in nature is controlled by sexual interactions across species. The interaction between a pheromone and its receptor is essential for successful mating and appropriate mate choice. In the *Saccharomyces* clade, outbreeding is thought to be relatively rare [47,48]; therefore, this hypothesis might be reasonable for yeasts in which the haploid is thought to be the stable point of their life cycle. The other hypothesis is that the farnesyl group of pheromones is the key determinant for mating partner discrimination. Whereas M cells have a high basal production of M-factor, P-factor production is fully dependent on M-factor stimulation [16,49]. Perhaps, this difference is related to the conservation of M-factor. Overall, it seems that the asymmetric system in *S*. *pombe* might allow flexible adaptation of the simple peptide to mutational changes in the pheromone/receptor pair while maintaining stringent recognition for mating partners by the lipid peptide, perhaps as a first step toward prezygotic isolation.

## Materials and methods

### Strains, media, and culture conditions

The strains constructed in this study are listed in S1 Table. The 150 wild *S*. *pombe* strains with different origins from the standard laboratory strain (Leupold’s strain, L968 [27]) were obtained from the National BioResource Project (deposited by J. Kohli, M. Sipiczki, G. Smith, H. Levin, A. Klar, and N. Rhind), H. Innan [28], and J. Bähler [29] (S2 Table).

Cells were grown in yeast extract (YE) medium supplemented with adenine (75 mg/l), uracil (50 mg/l), and leucine (50 mg/l). For solid medium, 1.5% agar was added to yeast extract agar (YEA). Where appropriate, antibiotics (G418, hygromycin B, and nourseothricin) were added to YEA plates at a final concentration of 100 μg/ml. EMM2 was also used for growth [50]. Synthetic Dextrose (SD) medium was used to select *S*. *pombe* auxotrophic mutants. Malt extract agar (MEA) medium, EMM2−N medium, and PMG medium were used for mating and sporulation [50,51]. Cells were grown and conjugated for a few days at 30 °C.

### Sequence analysis of wild strains

Genomic DNA was extracted from overnight cultures grown in YE medium. Each of the DNA fragments containing *mfm1*, *mfm2*, *mfm3*, *map2*, *map3*, *mam2*, and *sxa2* were amplified using the following primer sets: oTS83/84, oTS507/508, oTS509/510, oTS81/82, oTS91/92, oTS89/90, and oTS624/625, respectively (all primers are listed in S5 Table). The PCR products were sequenced using the internally specific primers: oTS504 (*mfm1*), oTS505 (*mfm2*), oTS506 (*mfm3*), oTS85/86 (*map2*), oTS157/158 (*map3*), oTS151/152 (*mam2*), and oTS647/648/658 (*sxa2*). The sequences obtained were compared with the corresponding sequence of L968. Differences from the L968 sequence are listed in S3 and S4 Tables.

### Construction of strains with variable numbers of P2 peptide in the Map2 ORF

The *map2*^+^ gene (approximately 2.6 kb) containing its promoter and terminator regions was amplified from L968 genomic DNA using the primer set oTS81/82. The DNA fragment was fused by using a Gibson Assembly (New England Biolabs) to a linearized vector derived from the integration vector pBS-ade6 [33], which was prepared by inverse PCR with the primer set oTS79/80. The resultant plasmid pTS13 (all plasmids used in this study are listed in S6 Table) was used as a starting template. To create a P2 unit, the correct two oligos (oTS73 and oTS93; reverse complements of each other) were mixed in Annealing Buffer (10 mM Tris-HCl, pH 7.4, 1 mM EDTA, and 50 mM NaCl), incubated at 70 °C for 10 minutes by a GeneAmp PCR System 9700, and then slowly cooled to 25 °C (approximately 1 hour). The DNA fragment that was amplified from the resulting double-strand DNA fragment (the P2 unit) using the primer set oTS74/75 was fused to a linearized vector derived from pTS13, which was prepared by inverse PCR with the primer set oTS71/72 to replace the *map2*^+^ gene. The resultant plasmid was referred to as pTS10.

To create Map2 ORFs with two to four P2 repeats, a further three P2 units carrying slightly different sequences at both sides (see S7 Fig for detailed sequence information about the *map2* genes constructed) were amplified from pTS10 using the appropriate primers (see S7 Table for all combinations of primers used for P-factor–related plasmids). Three mixed DNA fragments were fused to a linearized vector derived from pTS10, which was prepared by inverse PCR with the primer set oTS72/179. The resultant plasmids had various random repeats in the Map2 ORF. The number of repeats in each plasmid was checked by PCR using oTS85/86, and plasmids with the desired number of repeats (2–4 repeats) were sequenced to confirm their sequences. Thus, pTS47, pTS48, and pTS66 were constructed. The plasmids were cut near the center of the *ade6*^+^ gene with *Bam*HI and integrated at the *ade6* locus on Chromosome III in the FY23418 strain.

### Construction of strains producing various P-factor peptides

To generate pTS54, pTS55, pTS145, pTS146, and pTS233, inverse PCR was carried out using pTS10 and the primer sets oTS202/203, oTS204/205, oTS401/402, oTS403/404, and oTS204/404, respectively. The amplified PCR products were subjected to *Dpn*I treatment and 5′-phosphorylated by T4 polynucleotide kinase (TaKaRa), ligated by T4 ligase (TaKaRa), and transformed into *Escherichia coli* (DH5α). The introduced mutations were confirmed by sequencing the recovered plasmids. All plasmids carrying 4-repeat Map2 ORFs were then constructed, as described above.

To construct plasmids carrying two tandem *map2* genes with 4 repeats, the *map2* gene (approximately 2.6 kb) containing the promoter and terminator regions was amplified from the appropriate plasmid (pTS59, pTS48, pTS60, pTS147, pTS148, and pTS239) using the primer set oTS887/888. Each DNA fragment was fused to a linealized vector derived from the corresponding plasmid, which was prepared by inverse PCR with the primer set oTS885/886. Thus, pTS336–pTS341 were constructed.

In addition, the *map2* gene (approximately 2.6 kb) containing the promoter and terminator regions was amplified from the genomic DNA of nine wild strains (01CBS10391, 04CBS2775, 06CBS10460, 13CBS10504, 22CBS10468, 24CBS5682, 25CBS5680, 32CBS352, and 55FY28965) using the primer set oTS81/82. Each DNA fragment was fused to a linearized vector derived from the integration vector pBS-ade6, which was prepared by inverse PCR with the primer set oTS79/80. All of the obtained plasmids were integrated into the FY23418 strain, as described above.

### Quantitative assay of zygote formation

Cells grown on YEA plates overnight were resuspended in sterilized water to a cell density of 1 × 10^8^ cells/ml. A 30-μl aliquot of the resulting suspension was spotted onto sporulation media (MEA for *S*. *pombe*; PMG for *S*. *octosporus*) and then incubated for 2 days (*S*. *pombe*) or 3 days (*S*. *octosporus*) at 30 °C unless stated otherwise. The percentage of zygotes was calculated, as described previously [33,52]. In all cases, triplicate samples (at least 300 cells each) were counted, and the mean and standard deviation (SD) were calculated.

### Competitive test of hybrid formation by recombinant frequency

Heterothallic haploid strains each carrying a chromosomal drug-resistance marker (kanMX6, hphMX6, or natMX6) were cultured on YEA plates overnight, and the same cell numbers of the M-strain and two competing P-strains were mixed in sterilized water. A 30-μl aliquot of the resulting suspension was spotted onto MEA plate and incubated for exactly 24 hours at 30 °C. The mixed cells were allowed to mate, and the resulting hybrid diploids were sporulated to form spores. The cell suspension was diluted and spread on YEA plates containing different combinations of drugs. The number of colonies was counted after 3 days of incubation at 30 °C. The ratio of recombinant frequency was calculated, as described previously [11]. Three separate competitive tests were carried out.

### Shmooing assay

P-factor and M-factor peptides were chemically synthesized (Eurofins) for the shmooing assay. The purity of the preparations was over 95% (HPLC). P-factor was dissolved in dimethyl sulfoxide (DMSO) at a concentration of 500 μM, and M-factor was dissolved in methanol (MeOH) at a concentration of 1 mM. The stock solutions were diluted with culture medium to the appropriate dilution ratio. For the assay, heterothallic haploid cells were grown in YE medium overnight, washed with sterilized water three times, and then resuspended in EMM2−N medium at a cell density of 4 × 10^7^ cells/ml. The cells were treated with the synthetic pheromone and incubated for exactly 24 hours with gentle shaking.

To assess whether Mam2 could recognize different P-factors, a P-factor sensitive strain lacking the *sxa2*^+^ gene (TS402) was used. The cells were incubated with synthetic P-factors at different concentrations (0, 10, 100, and 1,000 nM) for 24 hours and observed by a DIC microscope. Images were recorded, and the L and W of a cell were measured to determine the L/W ratio. Cells with an L/W ratio of 2.0 or more were defined as shmooing cells; other cells were defined as arrested. In all cases, at least 100 cells each were measured. In the reciprocal experiment, an M-factor-sensitive strain lacking the *rgs1*^+^ gene [53] (TS405) was used to assess whether Map3 can recognize different M-factors. The cells were treated with synthetic M-factors at different concentrations (0, 100, and 1,000 nM) for 24 hours, and analyzed, as described above. An *sxa2Δrgs1Δ* double deletion strain (TS578) was used to accurately measure the activity of the P5 peptide.

### Preparation of culture medium from cells expressing Sxa2

The pREP vector was used to express *sxa2*^+^ under the control of the thiamine-repressible *nmt1*^+^ promoter in *S*. *pombe* [34]. First, a KanMX6 cassette amplified from pFA6a-kanMX6 [54] using the primer set oTS299/300 was fused to the linearized pREP1 vector prepared by inverse PCR with the primer set oTS301/302. In the resultant plasmid pTS111, the *LEU2* gene was replaced with a KanMX6 cassette. Next, the *sxa2*^+^ gene (approximately 1.5 kb) was amplified from L968 genomic DNA using the primer set oTS729/730 and fused to a linearized vector derived from the integration plasmid pTS111, which was prepared by inverse PCR with the primer set oTS305/306. The resultant plasmid pTS284 was transformed into TS402. Thus, a heterothallic strain (TS407) ectopically overexpressing *sxa2*^+^ under the control of the *nmt1*^+^ promoter was obtained.

The TS407 strain was precultured overnight in YE medium containing G418 and then washed with sterilized water three times. Cultures were inoculated into EMM2 medium containing G418 without thiamine (to induce the *nmt1*^+^ promoter) at a cell density of 1 × 10^7^ cells/ml and incubated with aeration for 2 days. The cell culture supernatant was passed through a 0.22-μm filter (Merck Millipore) to completely remove cell debris and then concentrated 20-fold by ultrafiltration through a Vivaspin 20-10K (GE healthcare) because ultrafiltration has been shown to concentrate the carboxypeptidase without loss of Sxa2 activity [21]. Thus, culture medium including active Sxa2 was obtained. As a control, pTS111 (*sxa2*^−^) was transformed into TS402, and the same preparation of culture medium was carried out.

### In vitro assay of the degradation level of P-factor

All reactions were performed at 30 °C in 50 mM citrate buffer, pH 5.5, with 200 μM synthetic P-factor as described previously [55]. Culture medium containing 100 ng of protein, as determined by a Bradford protein assay (BioRad), from strain TS406 or TS407 was added to a solution of P-factor, and then incubated for appropriate times (0, 10, and 60 mins) with gentle shaking via the constant-temperature incubator shaker MBR-022UP (TAITEC). Reactions were stopped by adding trifluoroacetic acid to 0.5%. The amount of leucine released from P-factor in the samples was measured by using a Branched Chain Amino Acid (BCAA) Assay kit (Cosmo Bio co.) in accordance with the manufacturer’s protocol and a Model 680 Microplate Reader (BioRad). Carboxypeptidase assays were performed in at least triplicates, and the mean ± SD was calculated. As a negative control, it was verified that almost no leucine was detected in an assay with a P-factor peptide lacking the C-terminal Leu residue (Table 2).

### Construction of strains producing pheromones from different species

Plasmid pTS189 was integrated at the *ade6* locus of an M-factorless strain (FY23412). The strain produced only So-M-factor and no Sp-M-factor. In contrast, the *So-map2*^+^ gene lacking both its signal sequence and two *N*-linked glycosylation sites was amplified from yFS286 genomic DNA using the primer set oTS57/58. The DNA fragment was fused to a linearized vector derived from pTS13, which was prepared by inverse PCR with the primer set oTS55/56 to replace the *map2*^+^ gene. The resultant plasmid pTS15 was integrated into the FY23418 strain, as described above. This strain produced only So-P-factors and no Sp-P-factors.

Next, we constructed an *S*. *octosporus* strain producing only Sp-P-factors. To delete the native *So-map2*^+^ gene, the 3′-downstream sequence (1 kb) was first amplified from yFS286 genomic DNA using the primer set oTS47/48. The DNA fragment was fused to a linearized vector derived from pFA6a-kanMX6, which was prepared by inverse PCR with the primer set oTS37/38 to construct the plasmid pTS5. Next, the 5′-downstream sequence (1 kb) was amplified from yFS286 genomic DNA using the primer set oTS46/78. The DNA fragment was fused to a linearized vector derived from pTS5, which was prepared by inverse PCR with the primer set oTS35/36, as described above. Lastly, pTS11 was constructed. The DNA fragment from this plasmid, namely, a 3.5-kb fragment amplified from pTS11 using the primer set oTS48/78, was purified, and 1 μg of the DNA was transformed into yFS286 by our previously described method [52]. The *So-map2*^+^ gene was successfully disrupted by a homologous recombination, and the resultant strain TS12 was sterile.

The *So-map2*^+^ gene (approximately 3.3 kb) containing the promoter and terminator regions was amplified from yFS286 genomic DNA using the primer set oTS78/626. The DNA fragment was fused to a linearized vector derived from the integration vector pFA6a-natMX6, which was prepared by inverse PCR with the primer set oTS35/36 to construct the plasmid pTS247. The *Sp-map2*^+^ gene lacking its signal sequence and two *N*-linked glycosylation sites was amplified from L968 genomic DNA using the primer set oTS635/636. The DNA fragment was fused to a linearized vector derived from pTS247, which was prepared by inverse PCR with the primer set oTS633/634 to replace the *So-map2*^+^ gene. The resultant plasmid pTS250 was integrated into the TS12 strain after restriction with *Bam*HI near the center of the terminator region. This strain produced only Sp-P-factors and no So-P-factors.

### Construction of strains expressing various *mam2* genes

The *mam2*^+^ gene (approximateley 3.1 kb) containing its promoter and terminator regions was amplified from L968 genomic DNA using the primer set oTS90/99. The DNA fragment was fused to a linearized vector derived from the integration vector pFA6a-hphMX6, which was prepared by inverse PCR with the primer set oTS35/36 to construct the plasmid pTS14. The *mam2* gene (approximately 1.0 kb) was amplified from the genomic DNAs of four wild strains (02CBS2628, 04CBS2775, 06CBS10460, and 26CBS5557) using the primer set oTS586/587. Each of the DNA fragments was fused to a linearized vector derived from pTS14, which was prepared by inverse PCR with the primer set oTS149/150 to replace the *mam2*^+^ gene. All obtained plasmids were integrated into the FY12677 strain after restriction with *Afe*I near the center of the terminator region.

### Construction of strains expressing various *sxa2* genes

The *sxa2*^+^ gene (approximately 3.5 kb) containing its promoter and terminator regions was amplified from L968 genomic DNA using the primer set oTS624/625. The DNA fragment was fused to a linearized vector derived from the integration vector pFA6a-natMX6, which was prepared by inverse PCR with the primer set oTS35/36 to construct the plasmid pTS246. The *sxa2* gene (approximately 1.5 kb) was amplified from the genomic DNAs of three wild strains (01CBS10391, 04CBS2775, and 101FY29038) using the primer set oTS664/665. Each of the DNA fragments was fused to a linearized vector derived from pTS246, which was prepared by inverse PCR with the primer set oTS627/628 to replace the *sxa2*^+^ gene. All obtained plasmids were integrated into the TS356 strain after restriction with *Bam*HI near the center of the terminator region.

## Supporting information

S1 Fig Phylogenetic tree of 151 *S*. *pombe* strains including the laboratory strain L968. (Related to Fig 1B.) The trees for the individual genes (*mfm1*, *mfm2*, *mfm3*, *map2*, *map3*, *mam2*, and *sxa2*) analyzed in this study are shown. mam, mating type auxiliary minus; map, mating type auxiliary plus; mfm, mating factor minus; sxa, sexually activated.
(TIFF)

S2 Fig Mating efficiency of various strains. (A) Zygote frequency of strains with a *map2* ORF containing different numbers of P-factor–encoding repeats: WT (4 repeats, L968), D9 (4 repeats, 24CBS5682), D13 (4 repeats, 55FY28965), D11 (5 repeats, 32CBS352), D1 (5 repeats, 01CBS10391), D5 (5 repeats, 06CBS10460), D8 (6 repeats, 22CBS10468), D3 (6 repeats, 04CBS2775), D10 (7 repeats, 25CBS5680), and D7 (8 repeats, 13CBS10504). (B) Zygote frequency of strains producing various P-factors (4 repeats). (C) Zygote frequency of strains expressing various *mam2* genes: WT (L968), F2 (04CBS2775), F1 (02CBS2628), F5 (26CBS5557), and F3 (06CBS10460). (D) Zygote frequency of strains expressing various *sxa2* genes or lacking the *sxa2*^+^ gene: WT (L968), G1 (01CBS10391), G4 (04CBS2775), and G7 (101FY29038). At least 300 cells were examined for each sample. Data are the mean ± SD of triplicate samples. The numerical data are included in S2 Data. Statistical significance was assessed by *t* test (*p < 0.05, ***p < 0.001). mam, mating type auxiliary minus; map, mating type auxiliary plus; n.s., not significant; ORF, open reading frame; P, Plus; sxa, sexually activated; WT, wild type.
(TIFF)

S3 Fig Shmooing assay of synthetic P-factor peptides. M cells lacking the *sxa2*^+^ gene (TS402) treated with synthetic P-factor at different concentrations (0, 10, 100, and 1,000 nM) were incubated in EMM2−N medium with gentle shaking for 24 hours. The ability of each P-factor peptide to induce shmooing was assessed by the L/W ratio of a cell. Cells with an L/W ratio of 2.0 or more were defined as shmooing cells (shown in black); those with a ratio of 2.0 less were defined as arrested cells (shown in white). Box-and-whisker plots reperesent the distribution of the L/W ratio; for each peptide, at least 100 cells each were measured. The numerical data are included in S2 Data. Scale bar, 5 μm. Significant differences between P2 and the other peptides at 10 nM was assessed by *t* test (P1−P2, *p* = 0.002; P3−P2, *p* = 0.047; P4−P2, *p* = 0.018; P5−P2, *p* < 0.001; P6−P2, *p* = 0.024). EMM2−N, Edinburgh Minimal Medium 2 lacking nitrogen; L, length; M, Minus; P, Plus; sxa, sexually activated; W, width.
(TIFF)

S4 Fig Shmooing assay of P5 peptide. To accurately measure of the activity of the P5 peptide, M cells doubly deleted for *sxa2*^+^ and *rgs1*^+^ (TS578) were treated with 1,000 nM synthetic P5 peptide and incubated in EMM2−N medium with gentle shaking for 24 hours. Box-and-whisker plots represent the distribution of the L/W ratio; at least 100 cells each were measured. The numerical data are included in S2 Data. Statistical significance was assessed by *t* test. EMM2−N, Edinburgh Minimal Medium 2 lacking nitrogen; L, length; M, Minus; n.s., not significant; rgs, regulator of G-protein signaling; sxa, sexually activated; W, width.
(TIFF)

S5 Fig Effects of mating pheromones of *S*. *octosporus* on *S*. *pombe* cells. (A) Comparison of the amino sequences of Sp-M-factor and So-M-factor. Identical amino acids are shown in gray, indicating that three of the nine amino acid residues (T2, V5, and Y7) of M-factor differ between the two species. (B) Shmooing assay of synthetic So-M-factor peptide and *S*. *pombe* cells. P cells lacking the *rgs1*^+^ (TS405) were treated with synthetic M-factor at different concentrations (0, 100, and 1,000 nM) and incubated in EMM2−N medium with gentle shaking for 24 hours. The ability of each M-factor peptide to induce shmooing was assessed by the L/W ratio of a cell, as described in S3 Fig. (C) Comparison of the amino sequences of Sp-P-factors and So-P-factors. Identical amino acids in all peptides are shown in gray, indicating that about eight of the 23 amino acid residues of P-factor differ between the two species. The amino acids that differ within each species are underlined in bold. (D) Shmooing assay of synthetic So-P-factor peptide and *S*. *pombe* cells. M cells lacking the *sxa2*^+^ (TS402) were treated with synthetic So-P-factor at different concentrations (0, 10, 100, and 1,000 nM) and incubated in EMM2−N medium with gentle shaking for 24 hours. The numerical data are included in S2 Data. The shmooing assay was evaluated as described in S3 Fig. EMM2−N, Edinburgh Minimal Medium 2 lacking nitrogen; L, length; M, Minus; P, Plus; rgs, regulator of G-protein signaling; So, *S*. *octosporus*; Sp, *S*. *pombe*; W, width.
(TIFF)

S6 Fig Morphology and mating frequency of cells expressing P1, P3, P4, and P6 peptides (with 4 repeats) during mating. (Related to Fig 2D.) For all strains carrying the *sxa2*^+^ gene, the cells form asci (arrows) containing four spores at a low frequency on MEA plates after 2 days. In the absence of Sxa2, the cells elongate more excessively, resulting in completely sterility. MEA, malt extract agar; sxa, sexually activated.
(TIFF)

S7 Fig Construction of *map2* genes with the Sp-P-factor–encoding sequence (4 repeats). Diagram showing the assembly of multiple DNA fragments containing a P-factor–encoding region with overlaps at the ends. The oligo primers used to amplify each DNA fragment, containing part of the specific sequence of spacers, are shown in S7 Table: spacers 1, 2, and 3 are indicated in red, green and blue, respectively. All modified nucleotide sequences encode the same amino acid sequences as those of the laboratory strain (L968). map; mating type auxiliary plus; P, Plus; Sp, *S*. *pombe*.
(TIFF)

S1 Table Strains used in this study. (XLSX)

S2 Table List of wild *S*. *pombe* strains whose origin differs from L968. (XLSX)

S3 Table Nucleotide sequence pattern of seven pheromone-associated genes in 150 wild *S*. *pombe* strains. (XLSX)

S4 Table Polymorphic alleles in the seven pheromone-associated genes of wild *S*. *pombe* strains. (XLSX)

S5 Table Primers used in this study. (XLSX)

S6 Table Plasmids used in this study. (XLSX)

S7 Table Combination of primers used for P-factor–related plasmids (4 peptide repeats). (XLSX)

S1 Data Text files including all nucleotide sequences of the seven pheromone-associated genes analyzed in 150 wild *S*. *pombe* strains and the laboratory strain L968. (ZIP)

S2 Data Excel files containing the underlying numerical data for Figs 2C, 2D, 2E, 3, 4C, 4D and 5; S2A, S2B, S2C, S2D, S3, S4, S5B and S5D Figs; and Table 2. (XLSX)

## Abbreviations

ABC: ATP-binding cassette
bar: barrier
EMM2−N: Edinburgh Minimal Medium 2 lacking nitrogen
GPCR: G-protein coupled receptor
krp: kexin-related protease
L: length
M: Minus
mam: mating type auxiliary minus
map: mating type auxiliary plus
MEA: malt extract agar
MEGA: Molecular Evolutionary Genetics Analysis software
mfm: mating factor minus
n.s.: not significant
nmt: no message in thiamine
ORF: open reading frame
P: Plus
So: *Schizosaccharomyces octosporus*
Sp: *Schizosaccharomyces pombe*
ste: sterile
sxa: sexually activated
W: width
WT: wild type
YE: yeast extract
YEA: yeast extract agar
