# Supplementary figures and images for: Asymmetric diversification of mating pheromones in fission yeast
Source: PLoS Biol. 2019 Jan 22;17(1):e3000101. doi: 10.1371/journal.pbio.3000101 (PMC6342294; doi:10.1371/journal.pbio.3000101)

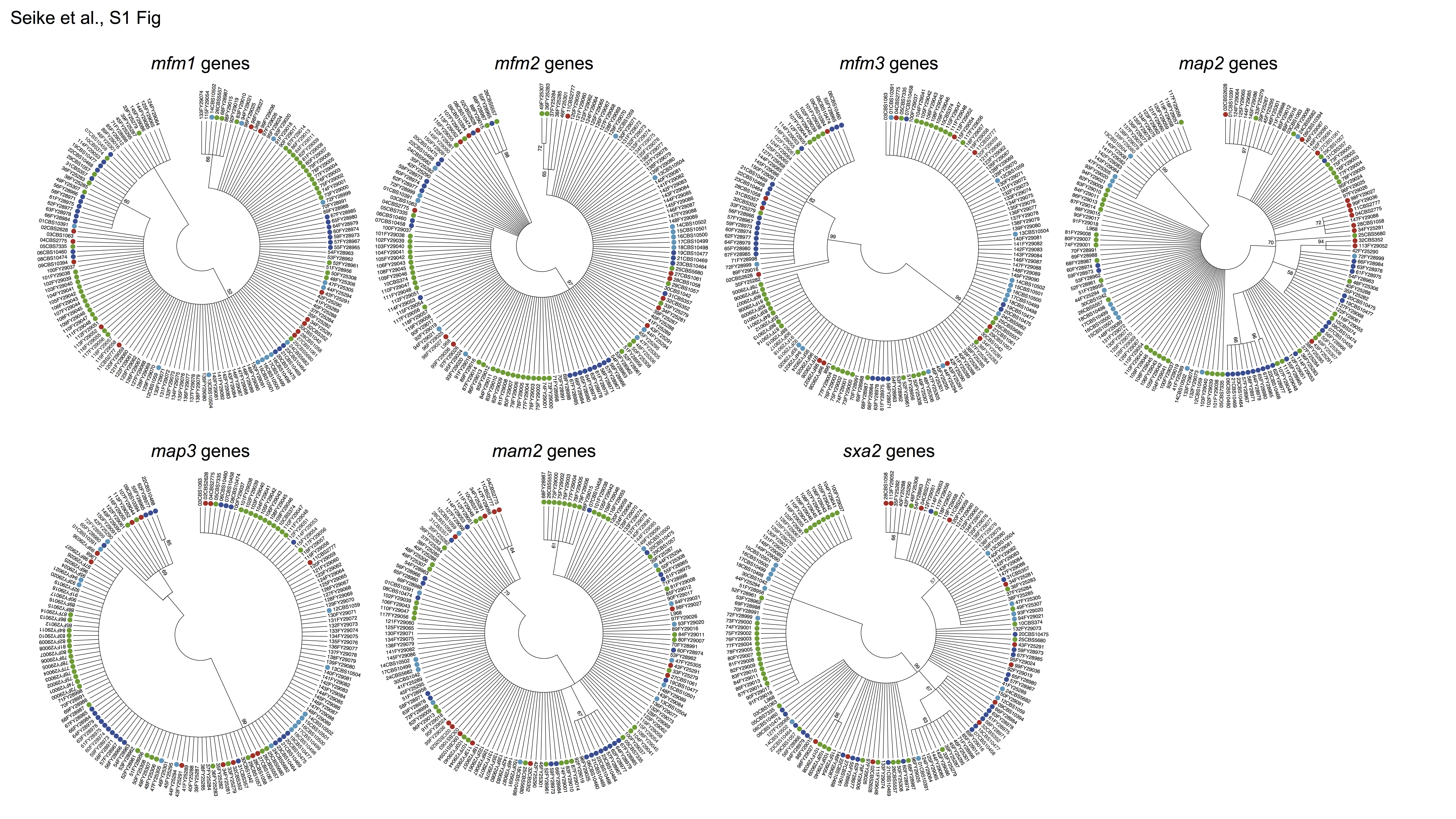

Supplement: S1 Fig — (Related to Fig 1B.) The trees for the individual genes (mfm1, mfm2, mfm3, map2, map3, mam2, and sxa2) analyzed in this study are shown. mam, mating type auxiliary minus; map, mating type auxiliary plus; mfm, mating factor minus; sxa, sexually activated. (TIFF) [file pbio.3000101.s001.tiff]

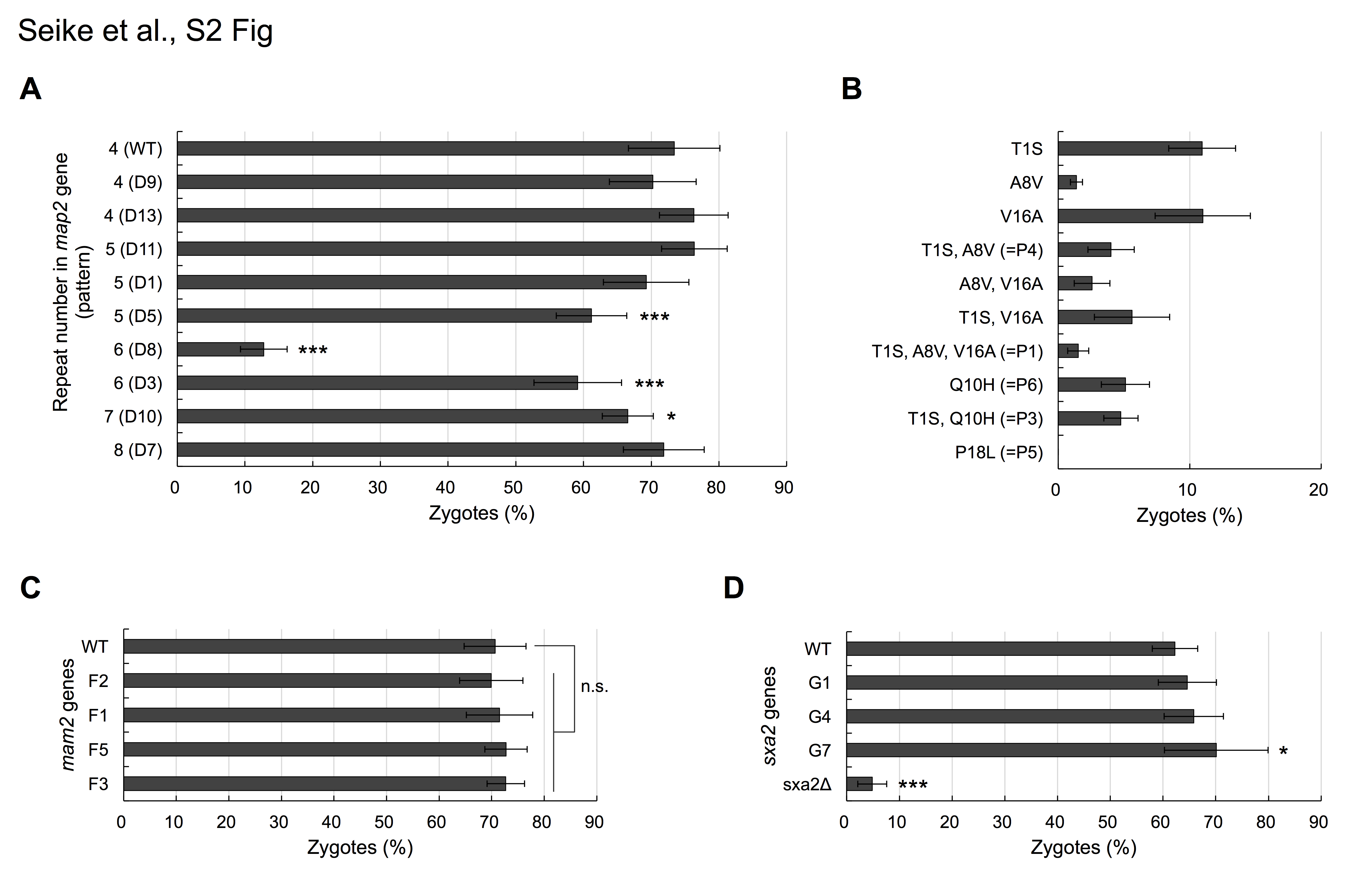

Supplement: S2 Fig — (A) Zygote frequency of strains with a map2 ORF containing different numbers of P-factor–encoding repeats: WT (4 repeats, L968), D9 (4 repeats, 24CBS5682), D13 (4 repeats, 55FY28965), D11 (5 repeats, 32CBS352), D1 (5 repeats, 01CBS10391), D5 (5 repeats, 06CBS10460), D8 (6 repeats, 22CBS10468), D3 (6 repeats, 04CBS2775), D10 (7 repeats, 25CBS5680), and D7 (8 repeats, 13CBS10504). (B) Zygote frequency of strains producing various P-factors (4 repeats). (C) Zygote frequency of strains expressing various mam2 genes: WT (L968), F2 (04CBS2775), F1 (02CBS2628), F5 (26CBS5557), and F3 (06CBS10460). (D) Zygote frequency of strains expressing various sxa2 genes or lacking the sxa2+ gene: WT (L968), G1 (01CBS10391), G4 (04CBS2775), and G7 (101FY29038). At least 300 cells were examined for each sample. Data are the mean ± SD of triplicate samples. The numerical data are included in S2 Data. Statistical significance was assessed by t test (*p < 0.05, ***p < 0.001). mam, mating type auxiliary minus; map, mating type auxiliary plus; n.s., not significant; ORF, open reading frame; P, Plus; sxa, sexually activated; WT, wild type. (TIFF) [file pbio.3000101.s002.tiff]

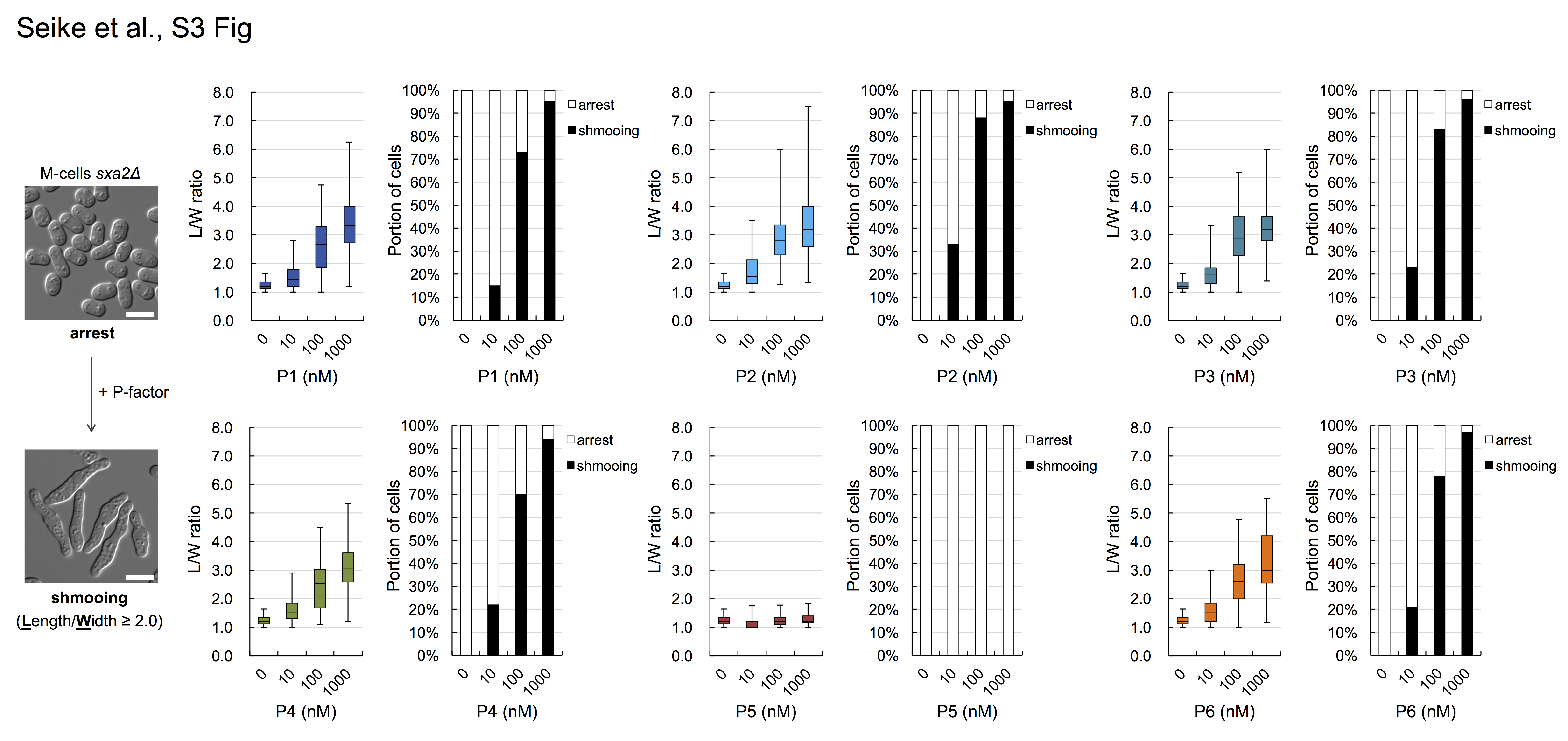

Supplement: S3 Fig — M cells lacking the sxa2+ gene (TS402) treated with synthetic P-factor at different concentrations (0, 10, 100, and 1,000 nM) were incubated in EMM2−N medium with gentle shaking for 24 hours. The ability of each P-factor peptide to induce shmooing was assessed by the L/W ratio of a cell. Cells with an L/W ratio of 2.0 or more were defined as shmooing cells (shown in black); those with a ratio of 2.0 less were defined as arrested cells (shown in white). Box-and-whisker plots reperesent the distribution of the L/W ratio; for each peptide, at least 100 cells each were measured. The numerical data are included in S2 Data. Scale bar, 5 μm. Significant differences between P2 and the other peptides at 10 nM was assessed by t test (P1−P2, p = 0.002; P3−P2, p = 0.047; P4−P2, p = 0.018; P5−P2, p < 0.001; P6−P2, p = 0.024). EMM2−N, Edinburgh Minimal Medium 2 lacking nitrogen; L, length; M, Minus; P, Plus; sxa, sexually activated; W, width. (TIFF) [file pbio.3000101.s003.tiff]

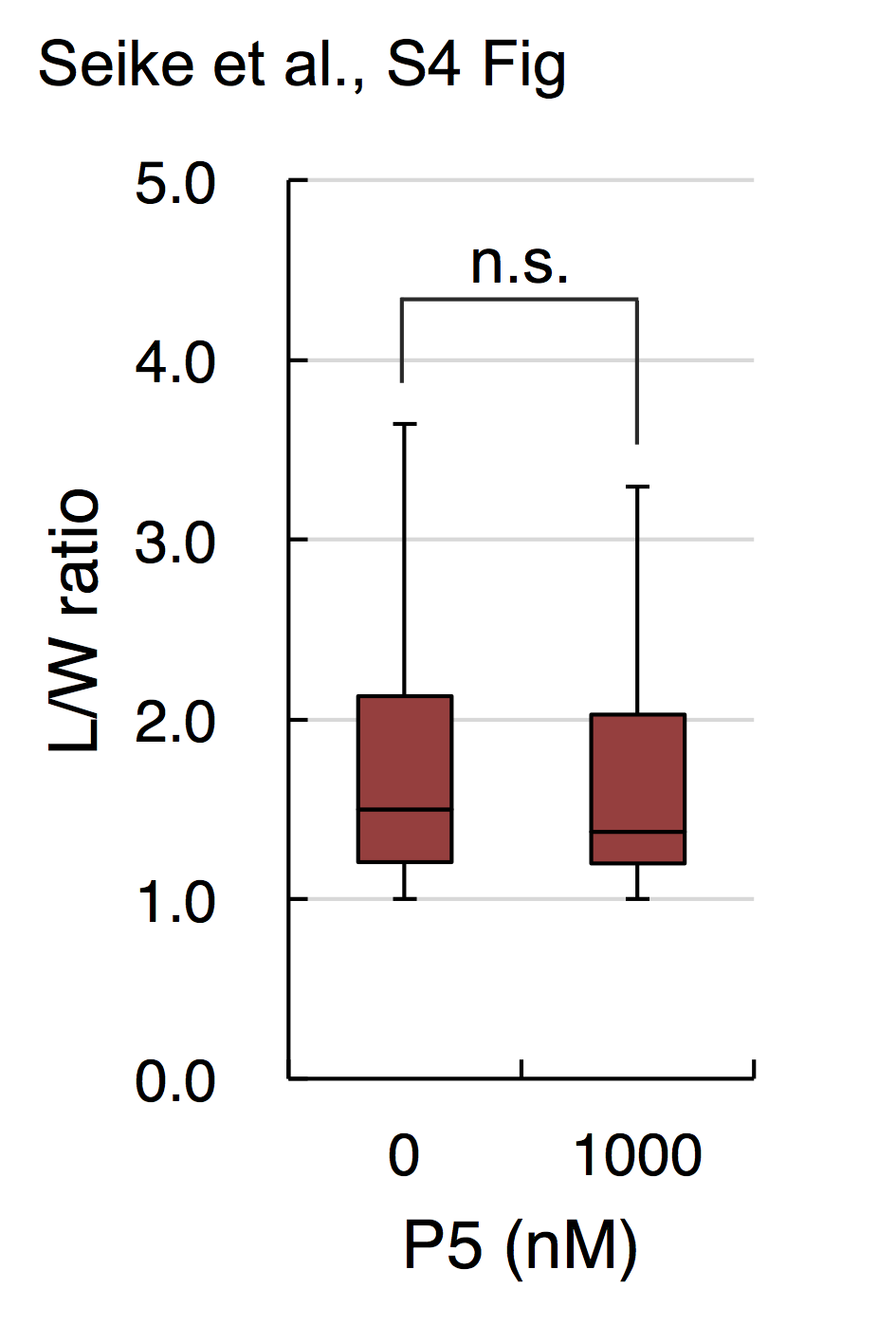

Supplement: S4 Fig — To accurately measure of the activity of the P5 peptide, M cells doubly deleted for sxa2+ and rgs1+ (TS578) were treated with 1,000 nM synthetic P5 peptide and incubated in EMM2−N medium with gentle shaking for 24 hours. Box-and-whisker plots represent the distribution of the L/W ratio; at least 100 cells each were measured. The numerical data are included in S2 Data. Statistical significance was assessed by t test. EMM2−N, Edinburgh Minimal Medium 2 lacking nitrogen; L, length; M, Minus; n.s., not significant; rgs, regulator of G-protein signaling; sxa, sexually activated; W, width. (TIFF) [file pbio.3000101.s004.tiff]

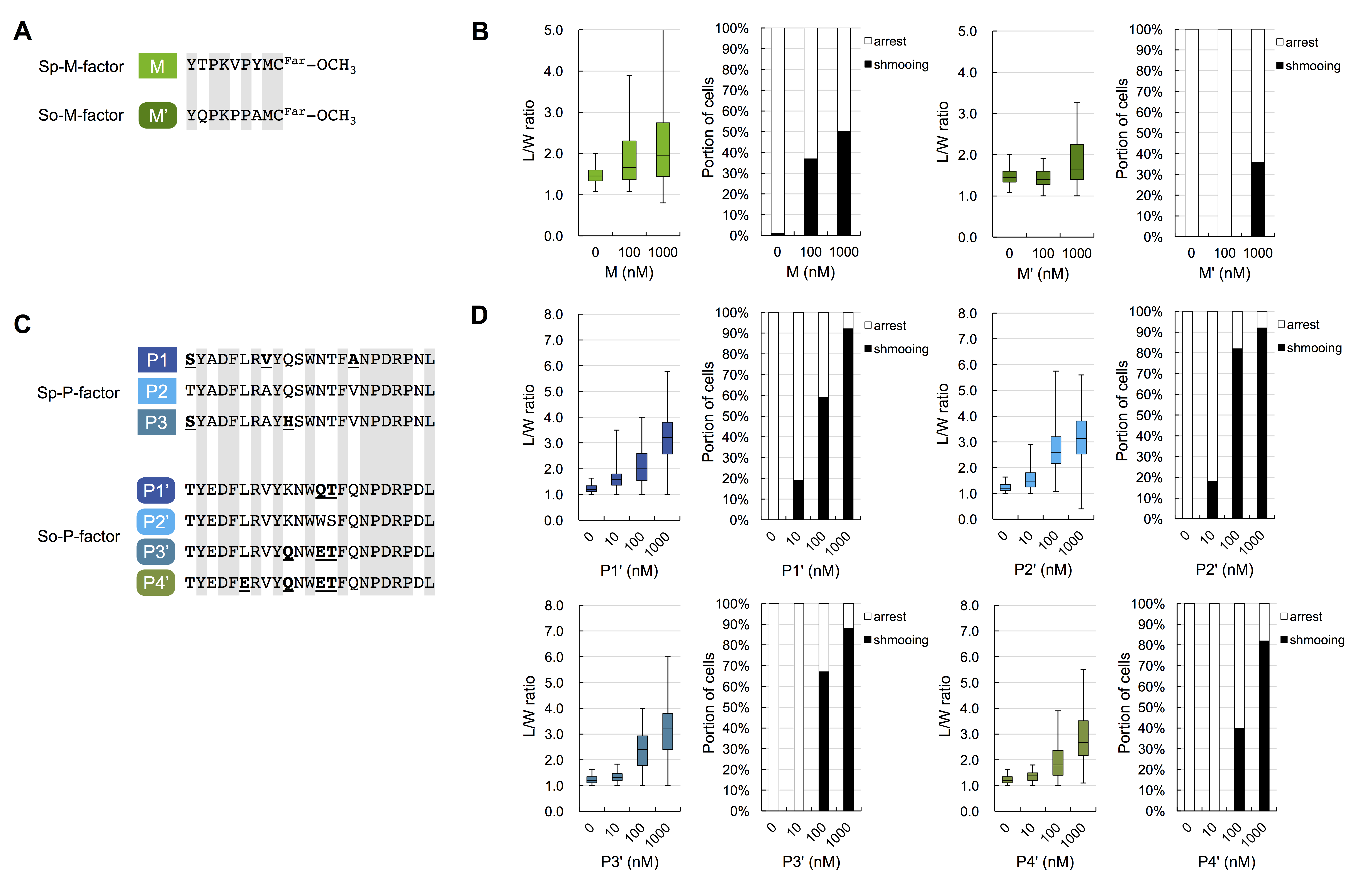

Supplement: S5 Fig — (A) Comparison of the amino sequences of Sp-M-factor and So-M-factor. Identical amino acids are shown in gray, indicating that three of the nine amino acid residues (T2, V5, and Y7) of M-factor differ between the two species. (B) Shmooing assay of synthetic So-M-factor peptide and S. pombe cells. P cells lacking the rgs1+ (TS405) were treated with synthetic M-factor at different concentrations (0, 100, and 1,000 nM) and incubated in EMM2−N medium with gentle shaking for 24 hours. The ability of each M-factor peptide to induce shmooing was assessed by the L/W ratio of a cell, as described in S3 Fig. (C) Comparison of the amino sequences of Sp-P-factors and So-P-factors. Identical amino acids in all peptides are shown in gray, indicating that about eight of the 23 amino acid residues of P-factor differ between the two species. The amino acids that differ within each species are underlined in bold. (D) Shmooing assay of synthetic So-P-factor peptide and S. pombe cells. M cells lacking the sxa2+ (TS402) were treated with synthetic So-P-factor at different concentrations (0, 10, 100, and 1,000 nM) and incubated in EMM2−N medium with gentle shaking for 24 hours. The numerical data are included in S2 Data. The shmooing assay was evaluated as described in S3 Fig. EMM2−N, Edinburgh Minimal Medium 2 lacking nitrogen; L, length; M, Minus; P, Plus; rgs, regulator of G-protein signaling; So, S. octosporus; Sp, S. pombe; W, width. (TIFF) [file pbio.3000101.s005.tif]

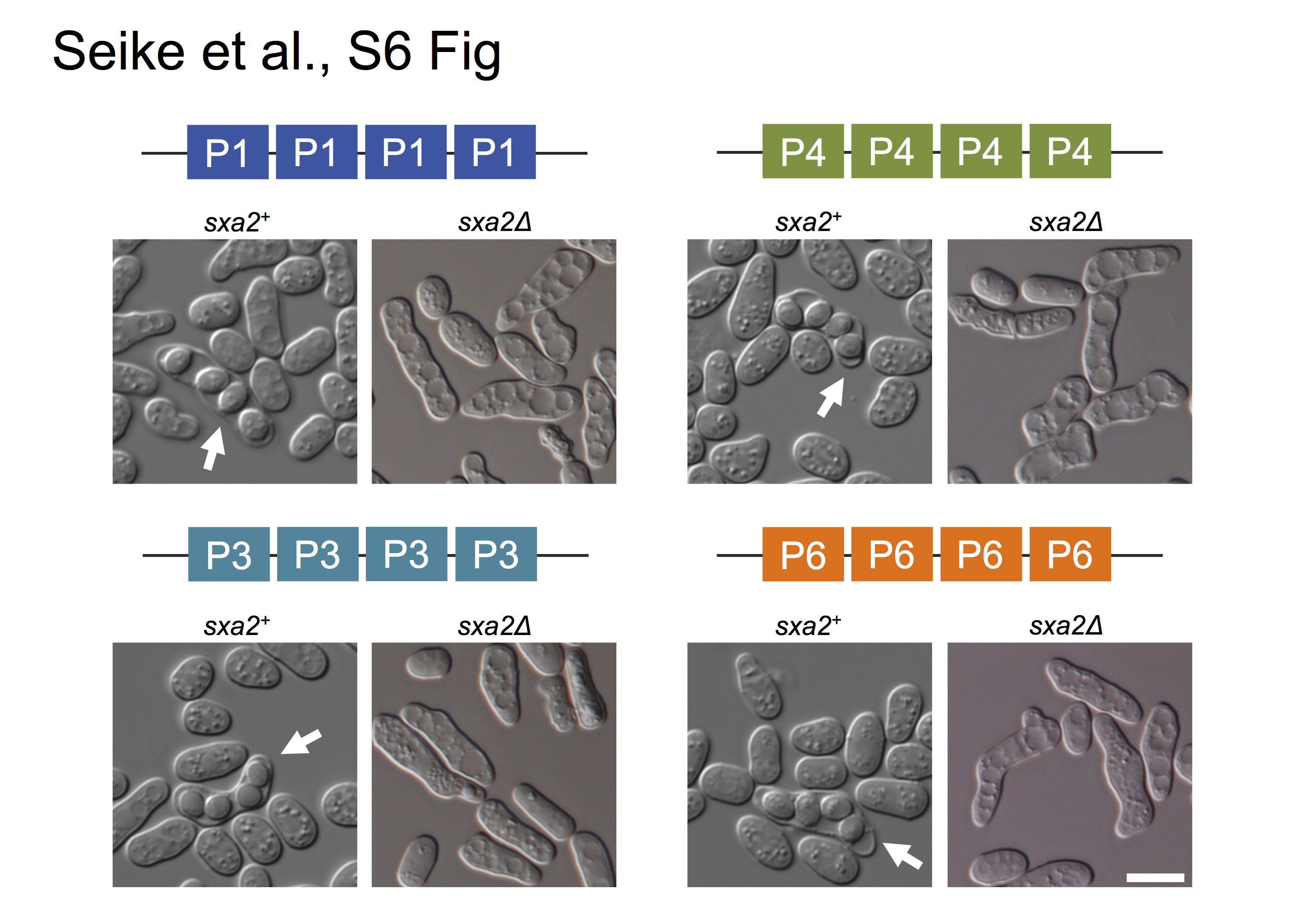

Supplement: S6 Fig — (Related to Fig 2D.) For all strains carrying the sxa2+ gene, the cells form asci (arrows) containing four spores at a low frequency on MEA plates after 2 days. In the absence of Sxa2, the cells elongate more excessively, resulting in completely sterility. MEA, malt extract agar; sxa, sexually activated. (TIFF) [file pbio.3000101.s006.tiff]

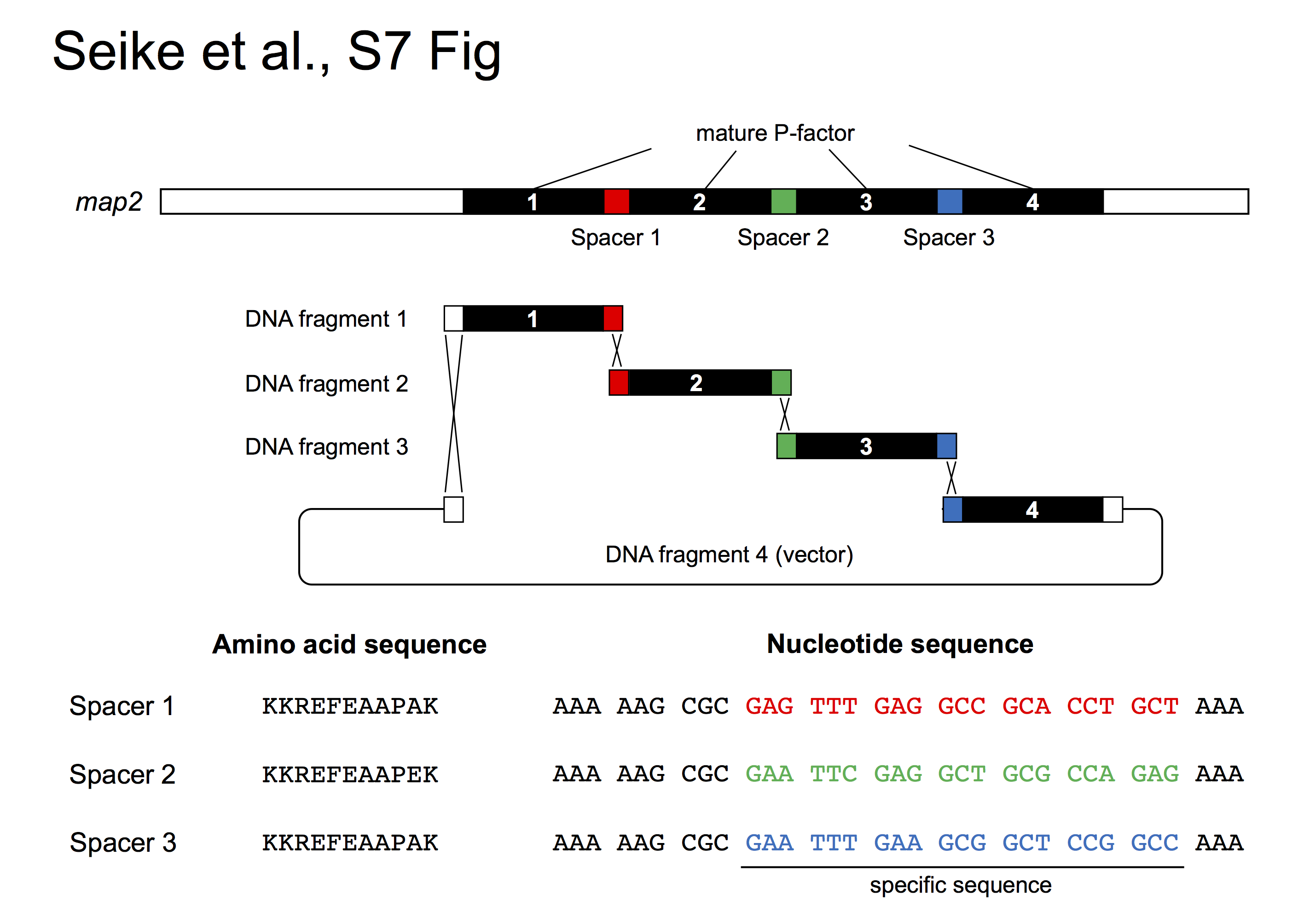

Supplement: S7 Fig — Diagram showing the assembly of multiple DNA fragments containing a P-factor–encoding region with overlaps at the ends. The oligo primers used to amplify each DNA fragment, containing part of the specific sequence of spacers, are shown in S7 Table: spacers 1, 2, and 3 are indicated in red, green and blue, respectively. All modified nucleotide sequences encode the same amino acid sequences as those of the laboratory strain (L968). map; mating type auxiliary plus; P, Plus; Sp, S. pombe. (TIFF) [file pbio.3000101.s007.tiff]
